# Mind the gap: trajectory of cognitive development in young individuals with sickle cell disease: a cross-sectional study

**DOI:** 10.3389/fneur.2023.1087054

**Published:** 2023-07-25

**Authors:** Melanie Koelbel, Shifa Hamdule, Fenella J. Kirkham, Hanne Stotesbury, Anna Marie Hood, Dagmara Dimitriou

**Affiliations:** ^1^Developmental Neurosciences Section, UCL Great Ormond Street Institute of Child Health, London, United Kingdom; ^2^Sleep Education and Research Laboratory, UCL Institute of Education, London, United Kingdom; ^3^Clinical and Experimental Sciences, University of Southampton, Southampton, United Kingdom; ^4^Division of Psychology and Mental Health, Manchester Centre for Health Psychology, University of Manchester, Manchester, United Kingdom

**Keywords:** sickle cell disease, cognition, development, executive function, age-related changes, cross-sectional study, verbal comprehension, silent cerebral infarction

## Abstract

**Study objectives:**

Compared to typically developing children and young adults (CYA-TD), those living with Sickle Cell Disease (CYA-SCD) experience more cognitive difficulties, particularly with executive function. Few studies have examined the relative importance of silent cerebral infarction (SCI), haemoglobin and arterial oxygen content on age-related cognitive changes using cross-sectional or longitudinal (developmental trajectory) data. This study presents cohort data from a single timepoint to inform studies with multiple timepoints.

**Methods:**

We compared cross-sectional raw and scaled scores as age-related changes in cognition (trajectories) in CYA-SCD and age-and ethnicity-matched CYA-TD. We also compared cross-sectional age-related changes in cognition (trajectories) in CYA-SCD with and without SCI to CYA-TD. General cognitive abilities were assessed using Wechsler Intelligence Scales, including the Verbal Comprehension Index (VCI) and Perceptual Reasoning Index (PRI) underpinning IQ. Executive function was evaluated using the Delis-Kaplan Executive Function System (D-KEFS) Tower subtest and the Behaviour Rating Inventory of Executive Function (BRIEF) questionnaire. SCI were identified from contemporaneous 3 T MRI; participants with overt stroke were excluded. Recent haemoglobin was available and oxygen saturation (SpO_2_) was measured on the day of the MRI.

**Results:**

Data were available for 120 CYA-SCD [62 male; age = 16.78 ± 4.79 years; 42 (35%) with SCI] and 53 CYA-TD (23 male; age = 17.36 ± 5.16). Compared with CYA-TD, CYA-SCD experienced a delayed onset in VCI and slower rate of development for BRIEF Global Executive Composite, Metacognition Index (MI), and Behaviour Regulation Index. The rate of executive function development for the BRIEF MI differed significantly between CYA-TD and CYA-SCD, with those with SCI showing a 26% delay compared with CYA-TD. For CYA-SCD with SCI, arterial oxygen content explained 22% of the variance in VCI and 37% in PRI, while haemoglobin explained 29% of the variance in PRI.

**Conclusion:**

Age-related cognitive trajectories of CYA-SCD may not be impaired but may progress more slowly. Longitudinal studies are required, using tests unaffected by practice. In addition to initiation of medical treatment, including measures to improve arterial oxygen content, early cognitive intervention, educational support, and delivery of extracurricular activities could support cognitive development for CYA-SCD.

**Graphical Abstract:**
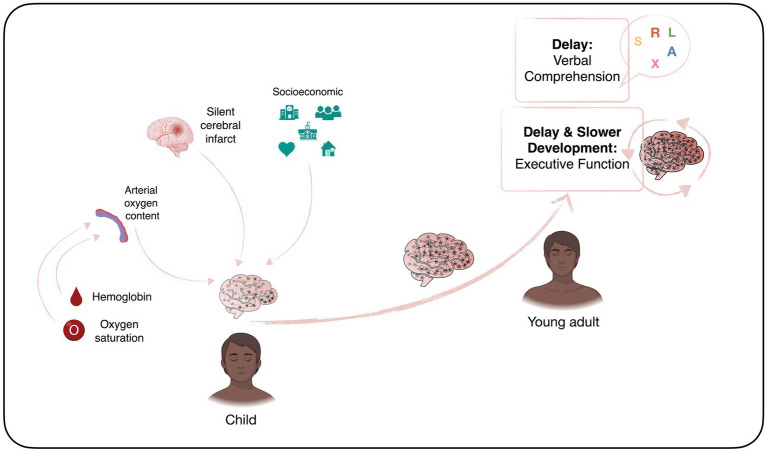

## 1. Introduction

**Figure 1:**
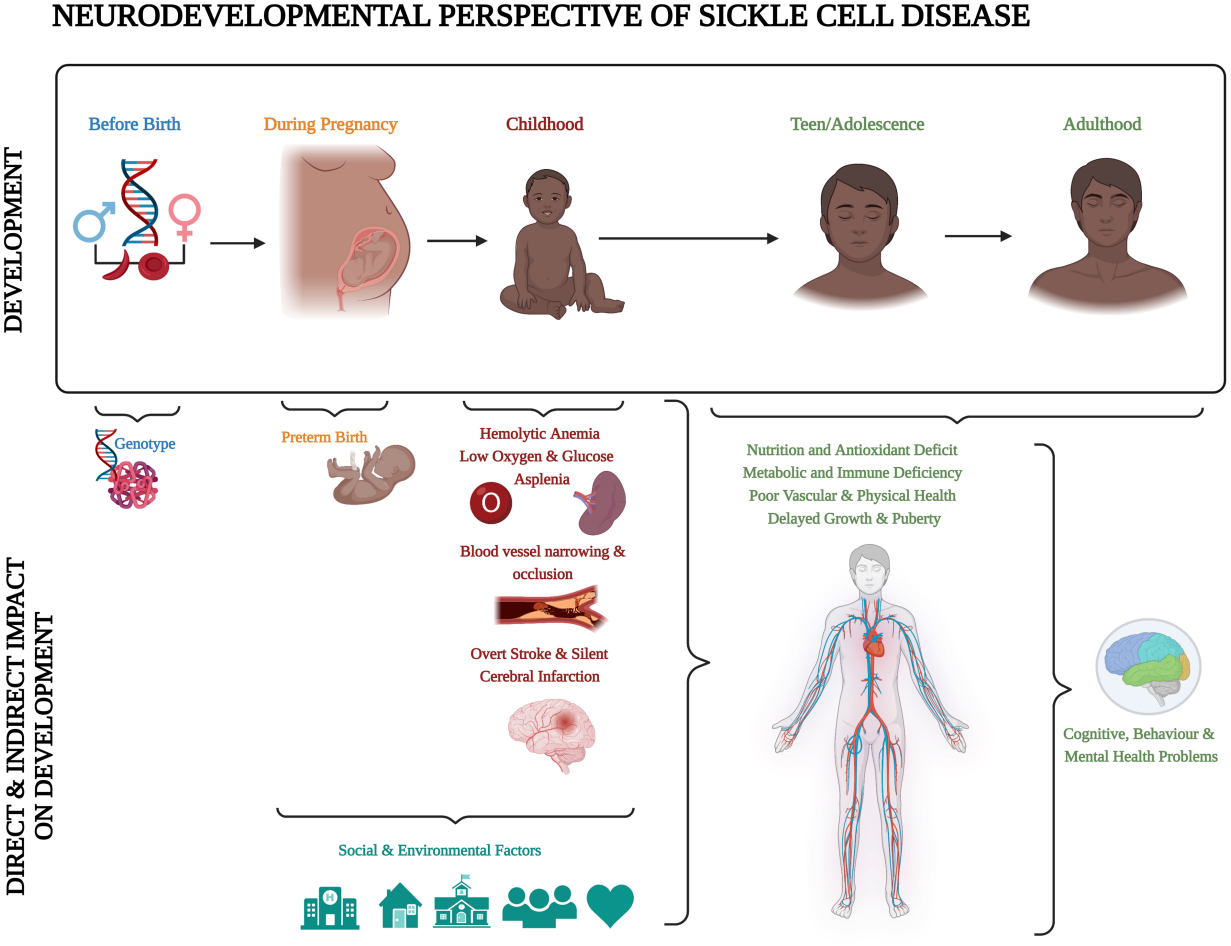
Neurodevelopmental perspective of sickle cell disease. Created with BioRender.com according to the information given in the review by Schatz and McClellan (4).

Sickle cell disease (SCD) is the most common inherited blood disorder, with about 275,000 babies born annually worldwide (1). In the homozygous form [sickle cell anaemia (SCA); HbSS], a combination of low haemoglobin and low oxygen saturation means that children are at risk of central nervous system complications, including stroke and seizures (2, 3). Accumulation of silent cerebral infarcts (SCI) on Magnetic Resonance Imaging (MRI) from an early age may result in cognitive difficulties that impact not only on academic attainment and life achievements, but also quality of life (4). Schatz and McClellan (4) identified SCD as a neurodevelopmental disorder because of the multifaceted impact of genes, vascular health, and social and environmental factors on early brain development (Figure 1). However, it is still unclear if individuals with SCD experience developmental delay, loss of skills related to brain injury after normal development, or atypical development due to a combination of both.

Previous research suggests that stroke and SCI contribute to the cognitive difficulties frequently seen in this population (5, 6). Systematic literature reviews have demonstrated that full-scale intelligence quotient (FSIQ) is lower than controls in individuals living with SCD, even those with no brain pathology (i.e., “normal MRI”: no SCI or stroke) (5, 7). However, cross-sectional data from studies using higher MRI field strengths have not found a robust association of SCI with cognition (8). As genetically determined and potentially modifiable environmental exposures may alter the development of cognition (9), there is a case for investigating cognitive profiles and trajectories in conditions where both are at play, such as SCD (10). To inform early interventions which might positively change the course of development, it is important to know when and why any difference(s) in development occur.

Full-Scale IQ (FSIQ) of adults or children may be measured with the Wechsler Intelligence Scales (11, 12), which include tests of different cognitive skills including the Verbal Comprehension Index (VCI), as well as Perceptual Reasoning (PRI), Working Memory (WMI) and Processing Speed Indices (PSI). The VCI subtests measure problem solving skills (e.g., subtest Similarities) (13, 14) and acquired general knowledge (e.g., Vocabulary) (14, 15). Executive Function (EF), including working memory, response selection (i.e., inhibition) and meta-tasking (16–18), is important for academic achievement and well-being in children. These cognitive domains develop at different stages throughout childhood from infancy to young adulthood (19), with multiple sensitive periods for each cognitive sub-domain (20). EF is typically measured using standardised tests, from batteries such as the Delis-Kaplan Executive Function System, or validated questionnaires such as the Behaviour Rating Inventory of Executive Function (BRIEF). Various socioeconomic factors (e.g., income-to-needs and maternal education) appear to be predictors of a child’s performance on EF tasks over time (21). Research has also shown that compromised health, such as is experienced in an inherited disease like SCD, might play an important role in the development of cognitive difficulties (22). Critically, children with SCD show early signs of reduced performance on measures of EF as well as general IQ (23, 24).

Previous research suggests that cognitive abilities are *impaired* in children and adults living with SCD and that difficulties are present at an early age (7). Most previous studies have only looked at cognitive abilities in specific age groups cross-sectionally. However, an approach which takes into account age-related changes is needed when studying neurodevelopmental disorders (25). Specific cognitive abilities (e.g., processing speed, EF) might impact on the early development of other cognitive abilities (26).

Wang et al. (27) reported cognitive data from 1 to 4 (mean 2.9 ± 1.0) timepoints in 467 children living with SCD (255 with HbSS) aged 6–18 years. At baseline those with HbSS and SCI had lower scores for FSIQ, Verbal IQ (VIQ) and Performance IQ (PIQ), as well as Digit Span, mathematics and reading achievement, at all ages. The hypothesis that there would be a decrease in psychometric performance with increasing age was addressed with follow-up data in between 122 and 173 children with HbSS and normal MRI only. It is important to note that during the study period, two versions of the Wechsler IQ scales were used with different scaling, so that age-related change, rather than longitudinal data, were reported for IQ. There was a significant decline in VIQ but not in PIQ or FSIQ with increasing age. On average VIQ declined with increasing age by 0.5 points/year (*p* < 0.04). After rescoring of the effectively equivalent subtests, longitudinal data could be reported for Working Memory (assessed using the Digit Span subtest) for which there was no evidence of decline, and Processing Speed (assessed using the Coding subtest) which declined 0.2 points per year (*p* < 0.01) (27). From the Woodcock-Johnson Tests of Achievement, there was a decline in performance for Mathematics (*p* < 0.01) but not for Reading. This study was conducted prior to the introduction of disease-modifying therapies, e.g., hydroxyurea. A Ghanaian study in adults aged 18–50 years with follow-up data after 7 years found a significant decline in verbal, non-verbal and global PIQ on the Revised Quick Cognitive Screening Test, as well as significant decreases in visuospatial abilities, processing speed, and EF (28). The rarity of these types of longitudinal evaluations in high and low resource countries is in part due to the logistical difficulties and expense.

An alternative to longitudinal evaluation is to examine existing cross-sectional databases to compare cognitive scores against age between typically developing children and young adults (CYA-TD) and those living with a long-term condition that may affect cognitive development. Although evaluating age-related changes in cross-sectional data is not truly a measure of developmental trajectory, it can help gain valuable insight in understanding developmental change over time to inform longitudinal studies. The assessment of age-related changes in cognitive development has been used in the previous studies of other developmental disorders (e.g., autism, Williams Syndrome and Down Syndrome) (29, 30), and was originally termed the developmental trajectory approach but has received little attention so far in children living with SCD (10, 31).

The approach described by Thomas et al. (32) helps to identify cognitive change with age and provides a more in-depth analysis of scores compared with simply reporting means across all ages. A significant linear relationship between age and the cognitive variable of interest in typically developing children is required to perform the developmental trajectory analysis in those with a long-term condition. Further analysis compares the two groups to identify if there might be a delay or slower rate of development in the non-typically developing population. The latter may be because there is a plateau or because there is no relationship at all with age, which can be distinguished by a rotational analysis (32).

Scaling to age-appropriate scores allows comparison with normative data, to see whether the groups develop differently as compared to their CYA-TD peers but might confound an analysis of age-related changes. Evaluation of raw scores is encouraged in longitudinal data as it provides a robust measure of individual change in the outcome measure, hence allowing evaluation of progress or decline for a particular individual across different time points. Exploring trajectories for raw as well as scaled scores might yield important insights when considering using cognitive endpoints in randomised clinical trials (33). Following this paradigm, we explored associations with both raw as well as scaled scores.

In this study, we investigated the cognitive development of children and young adults living with SCD (CYA-SCD) by adopting a cross-sectional age-related change (developmental trajectory) approach used in several previous studies (30, 32). The key aim was to investigate if there was a delay in the development of cognitive function, or if the rate of development differed, by comparing ethnicity and age-matched controls with patients living with SCD. Since previous literature has also showed an association between cognition and blood oxygenation measures [haemoglobin, peripheral oxygen saturation (SpO_2_) and arterial oxygen content (CaO_2_)] as well as SCI, we also aimed to investigate the effects of SCI, haemoglobin, SpO_2_ and CaO_2_ on cognitive development. Fox example, previous research found lower VIQ in adolescents with SCD and lower SpO_2_ (34), while lower processing speed was associated with lower CaO_2_ in CYA-SCD (35).

We hypothesised that:

1. CYA-SCD would perform worse on tests of intelligence and EF compared to age-and ethnicity-matched CYA-TD.
2. Developmental trajectories would be delayed, and the rate of development would be slower for CYA-SCD, with and without SCI, as compared to CYA-TD.
3. Factors explaining the cognitive differences between CYA-SCD and CYA-TD would include haemoglobin, SpO_2_ and CaO_2_.

## 2. Methods

### 2.1. Participants

Cross-sectional data were collected from Black CYA aged >8 years and ≤ 30 years with SCD and TD. A total of 173 Participants were enrolled in three different studies conducted at University College London: (1) The Sleep and Asthma Cohort (SAC) (36), (2) the Prevention of Morbidity in SCA 2b (POMS) (37), and (3) a community study in London which recruited through convenience and snowball sampling including advertisements on Twitter, Instagram, and in community centres and charities (38). For all three studies, inclusion criteria included the ability to speak English fluently and homozygosity for sickle cell haemoglobin (i.e., HbSS) or compound heterozygosity for sickle β thalassaemia zero (HbSβ0) for CYA-SCD. CYA-SCD were ineligible for SAC and POMS study participation if they were receiving nocturnal respiratory support at the time of enrolment, participating in a clinical trial evaluating blood transfusion or oxygen therapy, had chronic lung disease (other than asthma), or existing respiratory failure.

Additional exclusion criteria for the POMS study were hospital admissions within 1 month of enrolment or more than six hospital admissions within 12 months of enrolment (both for acute sickle-related complications), overnight oximetry showing mean overnight saturation of less than 90% for more than 30% of total sleep time, severe sleep apnoea defined by 4% oxygen desaturation index >15/h, and chronic blood transfusion or transfusion within 3 months of enrolment. The use of the disease-modifying therapy, hydroxyurea, was not an exclusion criterion. For the SAC study, patients were enrolled without regard to past sickle-or sleep-related morbidity or transfusion status. Black-British siblings, family members, peers of CYA-SCD, and people from the community in London, with no known neurological and psychological difficulties, were recruited as CYA-TD, whether they had sickle cell trait or SCI on MRI. Participants with or without SCD who had experienced an overt stroke were excluded. Each participant had a study protocol MRI on a Siemens 3 T PRISMA scanner (8). An independent neuroradiologist classified SCI according to the Silent Infarction Transfusion trial (39) (i.e., hyperintensity on FLAIR: >3 mm in diameter, present on two planes).

Detailed inclusion and exclusion criteria for individuals with SCD are explained elsewhere (36, 37, 40). West London NHS (SAC; 05/Q0408/42, 11/EM/0084, 15/LO/0347), Yorkshire NHS (POMS; 15/YH/0213), and University College London (14475/001) ethics committees provided ethical approval. Written informed consent was obtained from all participants and for children from their parent/guardian; the children also gave written assent.

### 2.2. Measures

#### 2.2.1. Questionnaires

Basic demographic information was obtained at the start of each study (i.e., age, sex, and postcode). The postcode-based Index of Multiple Deprivation was used as an indication of socioeconomic status (SES), as previously described (41). It consists of seven domains: income, employment, education, health, crime, barriers to housing and service, and living environment (42). The data are open-source, and downloadable from the UK Ministry of Housing, Communities & Local Government.^1^ The index ranges from rank 1st (most deprived) to 32,844th (least deprived).

#### 2.2.2. Cognitive assessment

General cognitive abilities were assessed using the Wechsler Intelligence Scale for Children (WISC-IV; SAC patients and CYA-TD < 16 years) (11), the Wechsler Adult Intelligence Scale (WAIS-IV; SAC patients and CYA-TD > 16 years) (12) or were estimated using the Wechsler Abbreviated Scale of Intelligence (WASI-II Vocabulary and Matrix Reasoning; POMS patients) (43). Scaled and raw scores for all indices and subtests were calculated to compare age-related changes. Scaled scores were derived from raw scores for each subtest, enabling comparison to the normative group based on the age range. Scaled scores from various subtests were combined together, resulting in indices for various domains of cognitive development: VCI (Verbal Comprehension: Similarities, Vocabulary, Information/Comprehension), PRI (Perceptual Reasoning: Block Design, Matrix Reasoning/Picture Concepts, Visual Puzzles), WMI (Working Memory: Digit Span, Arithmetic/Letter-Number-Sequencing), PSI (Processing Speed: Symbol Search, Coding/Cancellation) and FSIQ (Full-Scale IQ: sum of all raw scores). Raw scores have a mean of 10 and a standard deviation of 3. Scaled scores have a mean of 100 and a standard deviation of 15.

EF was assessed using the examiner-administered Tower subtest from the D-KEFS (44). Tower time was the outcome investigated, which has a mean of 10 and a standard deviation of 3. In addition, for a patient-centric measure, the caregiver-reported Behaviour Rating Inventory of Executive Function (BRIEF) (45) or adult self-reported BRIEF (BRIEF-A) were administered. The Global Executive Composite (GEC), Behaviour Regulation Index (BRI) and Metacognition Index (MI) and all subscale scores were investigated (46). The GEC is a summary index score of the BRI and MI. The BRI contains four different subscales: Inhibit, Shift, Emotional Control and Self-Monitor. The MI contains five subscales: Initiate, Working Memory, Plan/Organize, Task Monitor, and Organization of Materials. The caregiver-reported BRIEF does not contain the subscales of the BRI Self-Monitor and subscale for the MI Task Monitor. It also has an additional subscale the MI Monitor. T scores were used to interpret the BRIEF indices (normal < 60; mildly elevated: 60–64; potentially clinically elevated 65–69; clinically elevated above 70). Scaled and raw scores for all indices and subtests were calculated to compare developmental trajectories.

#### 2.2.3. Procedure

Participants completed their cognitive assessment on a single day in a quiet and comfortable room to minimise distractions. On the day of cognitive assessment, a fingertip pulse oximeter was used to measure daytime SpO_2_ (%). Haemoglobin (Hb in g/L) and Haematocrit (%) were obtained from the most recent laboratory test, partial pressure of oxygen (PaO_2_) was assumed to be 100 mmHg and arterial oxygen content (CaO_2_ in mL/dL) was calculated as


[SpO2×Hb(g/L)×1.37]+[PaO2(mmHg)×0.003]

### 2.3. Statistical analysis

Statistical analysis was performed using SPSS® version 26 (IBM Corporation, Armonk, NY, United States) for Mac^®^. Mean ± standard deviation (SD) was reported unless otherwise stated. For each variable, normality and homogeneity of variance were assessed using the Shapiro–Wilk test. Cook’s distance was used to identify potential outliers. Appropriate parametric (e.g., *t*-test, ANCOVA) or non-parametric (e.g., Mann–Whitney-U, Kruskal-Wallis) tests were then chosen to compare demographic and cognitive variables between CYA-SCD and CYA-TD, while controlling for age, sex, and SES. We investigated the relationship of haemoglobin, CaO_2_, SpO_2_ and SES in CYA-SCD on cognitive scores using simple linear regression models. We also inspected the scatter-and residual plots to check for linearity and outliers before assessing linear relationships.

### 2.4. Age-related change (developmental trajectory) analysis

To calculate and compare cross-sectional age-related changes (developmental trajectories), we used the method outlined by Thomas et al. (32), as summarised in the sections below. Detailed descriptions of this statistical approach have been published previously (30, 32) and used in cross-sectional studies of neurodevelopmental disorders (29, 30, 47). Further information is available at: http://www7.bbk.ac.uk/psychology/dnl/old_site/stats/Thomas_trajectories.html#Section2.

#### 2.4.1. Pre-analysis: simple linear regression

Trajectories can only be compared if there is a significant linear relationship between mental age and the cognitive variable in the CYA-TD group (32). To explore the importance of any differences in socioeconomic status across different cohorts, all models were corrected for SES. However, SES did not significantly influence the scores on cognitive tests. Hence, it was excluded from the ANCOVA analyses. For the analysis, mental age was calculated as (FSIQ score/100) * chronological age. Hence, the first step of our analysis was to generate simple linear regression models with each *scaled* cognitive variable as the outcome and mental age as the predictor for the CYA-TD group and the CYA-SCD group separately. Additionally, for *raw* scores, separate regression lines were calculated for WISC and WAIS data as the total raw scores for each subtest differ (See Supplementary Tables).

#### 2.4.2. Age-related changes (developmental trajectories) analysis

For our next step, we adjusted the age such that zero years represented the lowest age at which we collected data, i.e., 8 years. To do this, we deducted the lowest age from each participant’s mental age (30). Following this adjustment for age, we generated ANCOVA models that compared the developmental trajectory for cognitive scores only for those who showed a significant linear relationship with mental age in the CYA-TD group. The developmental trajectory analyses using ANCOVA resulted in two metrics: (1) if there was a *delay at onset*, i.e., if the two groups differed in performance at the youngest age at which the measurement is taken (also known as overall effect of the group), and (2) if the *rate of development* differed between the two groups which is the interaction between group performance and mental age (group × MA). It was also possible to calculate the difference between the two scores at onset by subtracting the intercept of the CYA-SCD group from the intercept of the CYA-TD group (*delay at onset* = intercept of CYA-TD group − intercept of CYA-SCD group). Similarly, difference in the rate of development is calculated by dividing the gradient of CYA-SCD group by that of the CYA-TD group (*rate of development* = gradient of CYA-SCD group/gradient of CYA-TD group).

#### 2.4.3. Rotational analysis (null trajectory analysis)

For cognitive domains where the CYA-TD group showed a significant relationship between scores and age, but the CYA-SCD group did not, we conducted a *rotation analysis* (X-Y coordinates) to distinguish between a *zero trajectory* and *no systematic relationship* in the CYA-SCD group, since results can be influenced by other factors such as cross-sectional design artifacts, missing disease severity data, and variability and static development in a particular cognitive domain [see detailed discussion in Thomas et al. (32)]. This allows distinction in the CYA-SCD group between plateauing (zero trajectory) or whether, alternatively, there is no systematic relationship, suggesting that there might be other variables that predict the relationship better.

Using the same approach, we also examined if developmental trajectories differed between CYA-SCD with and without SCI.

## 3. Results

### 3.1. Participant characteristics

Our study included 120 CYA-SCD participants [Genotypes: HbSS: 99%, *N* = 119; HbSB0-thal: 1%, *N* = 1, 62 males; mean age = 16.78 ± 4.79; age range 8–30 years; *N* = 53 (44%) ≤ 16 years]. Fifty-three CYA-TD [34% HbAS; 23 males; mean age = 17.36 ± 5.16; age range 8.2–30 years; *N* = 23 (43%) ≤ 16 years] were eligible for inclusion in the developmental trajectory analysis (Table 1). Just over one third of CYA-SCD had SCI (Table 1). No significant differences for age, sex, or SES (*p* > 0.05) were observed between CYA-SCD and CYA-TD, or for CYA-SCD with (*N* = 49) or without hydroxyurea prescription (*N* = 71). CYA-SCD had significantly lower SpO_2_, CaO_2_, haemoglobin and haematocrit compared to CYA-TD (Table 1).

**Table 1:** Participant comparison.

	*N*	CYA-SCD	CI 95%	*N*	CYA-TD	CI 95%	*p* ^1^	Effect size	*p* ^2^	Effect size
Demographics										
Gender	120	62 Male		53	23 Male		> 0.05			
		58 Female			30 Female					
MRI (*N*), SCI (%)	106	42, 35%			46, 10%					
Genotype (*N*)		HbSS (119)			HbAA (35)					
		HbSB0-thal (1)			HbAS (18)					
Age in years	120	16.78 (4.79)	[15.91, 17.65]	53	17.36 (5.16)	[15.94, 18.78]	> 0.05*	0.12		
		8.02–29.40			8.24–30.67					
SES	115	9995.15 (6541.42)	[8786.76, 11205.54]	53	9322.94 (6537.98)	[7520.85, 11125.03]	> 0.05*	0.14		
		1.443–32.371			3.056–32.371					
SpO_2_%	55	96.41 (3.41)	[95.50, 97.34]	39	98.56 (1.43)	[98.10, 99.03]	< 0.001*	0.75	0.001	0.11
		86.91–100			93–100					
CaO_2_ mL/d	106	11.73 (1.93)	[11.36, 12.10]	47	18.13 (1.04)	[17.82, 18.43]	< 0.00*	2.59	0.001	0.73
		7.79–17.55			16.86–20.68					
Hemoglobin g/L	106	88.30 (14.23)	[85.30, 90.78]	47	134.89 (8.55)	[132.38, 137.41]	< 0.00*	2.51	< 0.001	0.73
		60–134			126–152					
Hematocrit %	106	26.34 (4.57)	[25.46, 27.22]	47	39.60 (5.91)	[37.86, 41.34]	< 0.00*	2.23	< 0.001	0.57
		16.10–40			3.4–45.3					
Wechsler cognitive scores										
FSIQ	120	93.16 (13.07)	[90.80, 95.52]	53	98.43 (12.10)	[95.10, 101.77]	0.01	0.41	0.006	0.05
		58–123			75–130					
VCI	69	95.48 (13.71)	[92.19, 98.77]	53	99.40 (11.72)	[96.17, 102.63]	0.09	0.30	0.07	0.03
		57–138			71–130					
Similarities	69	9 (2.72)	[8.35, 9.65]	53	10.17 (2.23)	[9.56, 10.78]	0.01	0.46	0.02	0.05
		3–17			6–16					
Vocabulary	120	9.2 (2.90)	[8.68, 9.72]	53	10.11 (2.73)	[9.36, 10.87]	0.10*	0.25	0.015	0.04
		2–17			5–17					
PRI	69	91.07 (10.79)	[88.48, 93.66]	53	96.94 (10.53)	[94.04, 99.85]	0.003	0.55	0.003	0.08
		61–111			73–117					
Block Design	69	7.54 (2.18)	[7.01, 8.06]	53	8.43 (2.21)	[7.83, 9.04]	0.02*	0.44	0.03	0.04
		3–14			4–13					
Matrix Reasoning	120	9.62 (2.82)	[9.11, 10.13]	53	10.60 (2.66)	[9.87, 11.34]	0.03*	0.33	0.01	0.04
		1–16			4–15					
WMI	120	92.71 (13.70)	[90.23, 95.19]	53	100.68 (13.18)	[97.05, 104.31]	0.001	0.59	< 0.001	0.07
		56–136			65–136					
Digit Span	120	8.84 (2.57)	[8.38, 9.31]	53	10.60 (2.73)	[9.85, 11.36]	< 0.001*	0.62	< 0.001	0.08
		3–17			5–19					
Arithmetic	114	8.54 (3.04)	[7.97, 9.10]	49	10.16 (2.73)	[9.38, 10.95]	0.001*	0.52	0.001	0.07
		2–19			5–18					
PSI	120	89.25 (13.28)	[86.85, 91.65]	53	98.51 (14.29)	[94.57, 102.45]	< 0.001	0.68	< 0.001	0.11
		59–131			53–122					
Coding	120	7.77 (2.65)	[7.29, 8.25]	53	9.42 (3.04)	[8.58, 10.25]	< 0.001*	0.54	< 0.001	0.08
		2–16			2–15					
Symbol Search	120	8.43 (3.01)	[7.88, 8.97]	53	9.64 (2.92)	[8.84, 10.45]	0.01*	0.41	0.01	0.04
		1–15			1–15					
Cancellation	120	9.38 (3.60)	[8.72, 10.03]	52	10.29 (2.89)	[9.49, 11.09]	0.08	0.27	0.10	0.02
Executive function scores										
BRIEF GEC	95	52.44 (10.79)	[50.24, 54.64]	39	50.67 (11.08)	[47.07, 54.26]	0.40	−0.16	0.38	0.01
		34–80			33–78					
BRIEF BRI	95	52.04 (10.92)	[49.82, 54.27]	39	51.59 (11.13)	[47.98, 55.20]	0.85*	0.03	0.88	0.00
		34–84			34–75					
Inhibit	67	51.72 (11.54)	[48.90, 54.53]	39	51.82 (10.75)	[48.34, 55.31]	0.84*	0.04	0.72	0.001
		36–103			36–87					
Shift	67	52.75 (11.87)	[49.85, 55.64]	39	52.77 (10.75)	[49.29, 56.25]	0.87*	0.03	0.79	0.001
		38–81			38–73					
Emotional Control	66	50.76 (11.99)	[47.81, 53.70]	39	51.56 (12.97)	[47.36, 55.77]	0.83*	0.04	0.63	0.002
		37–80			37–83					
BRIEF MI	96	52.66 (10.81)	[50.47, 54.85]	39	50.41 (11.55)	[46.67, 54.15]	0.19*	0.23	0.27	0.01
		33–82			34–85					
Initiate	67	53.61 (11.36)	[50.84, 56.38]	39	49.36 (11.01)	[45.79, 52.93]	0.08*	0.34	0.10	0.03
		35–79			35–69					
Working Memory	67	56.31 (12.88)	[53.17, 59.45]	39	52.23 (9.96)	[49.00–55.46]	0.14*	0.29	0.16	0.02
		38–89			38–71					
Plan/Organize	67	54.07 (11.24)	[51.33, 56.82]	39	49.36 (10.20)	[46.05, 52.66]	0.04*	0.42	0.07	0.03
		38–86			37–74					
Organise Material	67	49.97 (9.97)	[47.54, 52.40]	39	47.74 (8.19)	[45.09, 50.40]	0.31*	0.20	0.48	0.01
		34–71			34–67					
Delis-Kaplan Tower Time	114	556.67 (145.05)	[529.75, 583.58]	50	563.74 (146.25)	[522.18, 605.39]	0.78	0.05	0.73	0.001

Results are given in mean (±SD) and range. *p value for Mann–Whitney U test. *p*^1^, mean difference without confounding variables. *p*^2^, mean difference with confounding variables (age, sex, and SES). CYA-SCD, children and young adults living with sickle cell disease; CYA-TD, children and young adults who are typically developing; MRI, Magnetic resonance imaging; SCI, silent cerebral infarcts; SES, Socioeconomic status; SpO_2_, Oxygen saturation; CaO_2_, arterial oxygen content; FSIQ, Full Scale IQ; VCI, Verbal Comprehension Index; PRI, Perceptual Reasoning Index; WMI, Working Memory Index; PSI, Processing Speed Index; BRIEF, Behaviour Rating Inventory of Executive Function; BRIEF GEC, Global Executive Composite; BRIEF BRI, Regulation Index; BRIEF MI, Metacognition Index.

### 3.2. Cognitive profile

After controlling for age, sex and SES, there were significant mean group differences between CYA-SCD and CYA-TD for IQ, VCI Similarities and Vocabulary, PRI and PRI Block Design and Matrix Reasoning, WMI and WMI Digit Span and Arithmetic, PSI and PSI Coding and Symbol Search but no significant differences for variables measuring EF (Table 1).

After controlling for age, sex and SES, there were no significant mean group differences in demographics, for CYA-SCD with (CYA-SCD-SCI+) and without (CYA-SCD-SCI−) silent cerebral infarct (Supplementary Tables). Significant group differences for CYA-SCD-SCI+ and CYA-TD were found for PRI and PRI Block Design, WMI and WMI Digit Span, PSI and PSI Symbol Search and Cancellation. Significant group differences for CYA-SCD-SCI+, CYA-SCD-SCI− and CYA-TD were found for PRI, WMI and WMI Digit Span and Arithmetic, PSI and PSI Coding and Cancellation. No significant differences were found for variables measuring EF. All values were controlled for age, sex and SES and are available in Supplementary Table S1.

### 3.3. Developmental trajectories (age-related changes) for scaled cognitive scores

The results for scaled scores developmental trajectories are presented below for each step of the analysis, with the data on delay at onset and rate of development for CYA-SCD compared with CYA-TD presented in Table 2.

**Table 2:** Developmental trajectories for scaled scores.

Variable	Slope: cognitive variable = (intercept for group) + (age * gradient)		Delay at onset	*p*	CI 95%	Rate of development	*p*	CI 95%
	CYA-SCD	CYA-TD	CYA-SCD compared with CYA-TD					
General cognitive scores								
VCI	81.79 + age * 1.48	91.05 + age * 0.8	9.26	0.05	[0.219, 18.311]	1.85	0.11	[−1.491, 0.147]
Executive function scores								
BRIEF GEC	53.53 + age * −0.07	33.03 + age * 1.03	−20.5	0.003	[−21.48, −4.58]	−0.07	0.005	[0.028, 0.156]
BRIEF BRI	57.5 + age * −0.36	37.38 + age * 0.83	−20.12	0.004	[−20.66, −3.50]	−0.43	0.003	[0.034, 0.164]
BRI Inhibit	53.38 + age * −0.1	33.32 + age * 1.08	−20.06	0.01	[−21.56, −2.56]	−0.09	0.01	[0.029, 0.168]
BRIEF MI	50.73 + age * 0.13	30.59 + age * 1.16	−20.14	0.003	[−21.61, −4.65]	0.11	0.01	[0.022, 0.150]
MI Initiate	52.48 + age * 0.07	32.09 + age * 1.01	−20.39	0.01	[−23.55, −4.45]	0.07	0.03	[0.009, 0.149]
MI Working Memory	63.39 + age * −0.42	35.43 + age * 0.99	−27.96	<0.001	[−28.51, −8.35]	−0.42	0.002	[0.044, 0.191]
MI plan/organise	58.69 + age * −0.28	36.79 + age * 0.74	−21.9	0.01	[−24.42, −5.66]	−0.38	0.03	[0.016, 0.153]

Delay at onset = intercept of CYA-TD group – intercept of CYA-SCD group; rate of development = gradient of CYA-SCD group/gradient of CYA-TD group. CYA-SCD, children and young adults living with sickle cell disease; CYA-TD, children and young adults who are typically developing; VCI, Verbal Comprehension Index; BRIEF GEC, Global Executive Composite; BRIEF BRI, Regulation Index; BRIEF MI, Metacognition Index; BRIEF, Behaviour Rating Inventory of Executive Function.

#### 3.3.1. Pre-analysis: simple linear regression

Cross-sectional developmental trajectory analyses explored cognitive profiles in CYA-SCD in comparison to CYA-TD. SES did not significantly influence any of the cognitive scores and was excluded from further analyses. All simple linear regression results examining the relationships between mental age and performance on cognitive scores are shown in Supplementary Table S2. To compare the groups in the later steps of the analysis, the pre-analysis (simple linear regression) required a statistically significant result for the CYA-TD group (Supplementary Table S2) which was the case for VCI, the VCI Similarities and Vocabulary subtests, PRI, the PRI Block Design subtest and WMI as well as the PSI Coding subtest. For the measures of EF the BRIEF GEC was close to being significantly related to mental age. The BRIEF MI and its subscales Initiate, Working Memory, and Plan/Organize as well as the BRIEF BRI and its subscale Inhibit, all showed a significant relationship with mental age (see Supplementary Table S2).

#### 3.3.2. Cross-sectional developmental trajectory analysis

**Figure 2:**
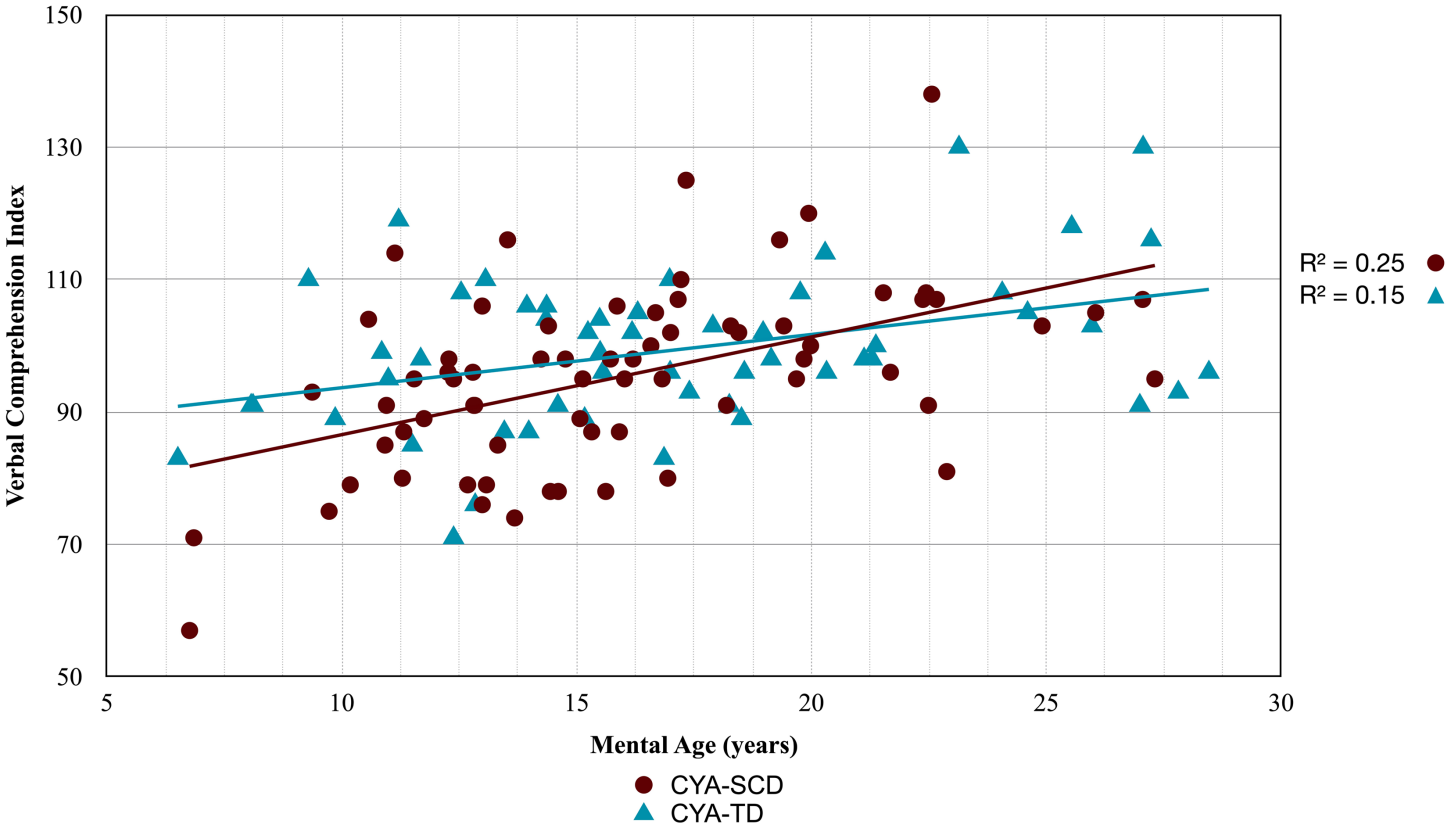
Developmental trajectory of Verbal Comprehension Index. CYA-SCD, children and young adults with sickle cell disease; CYA-TD, children and young adults who are typically developing.

**Figure 3:**
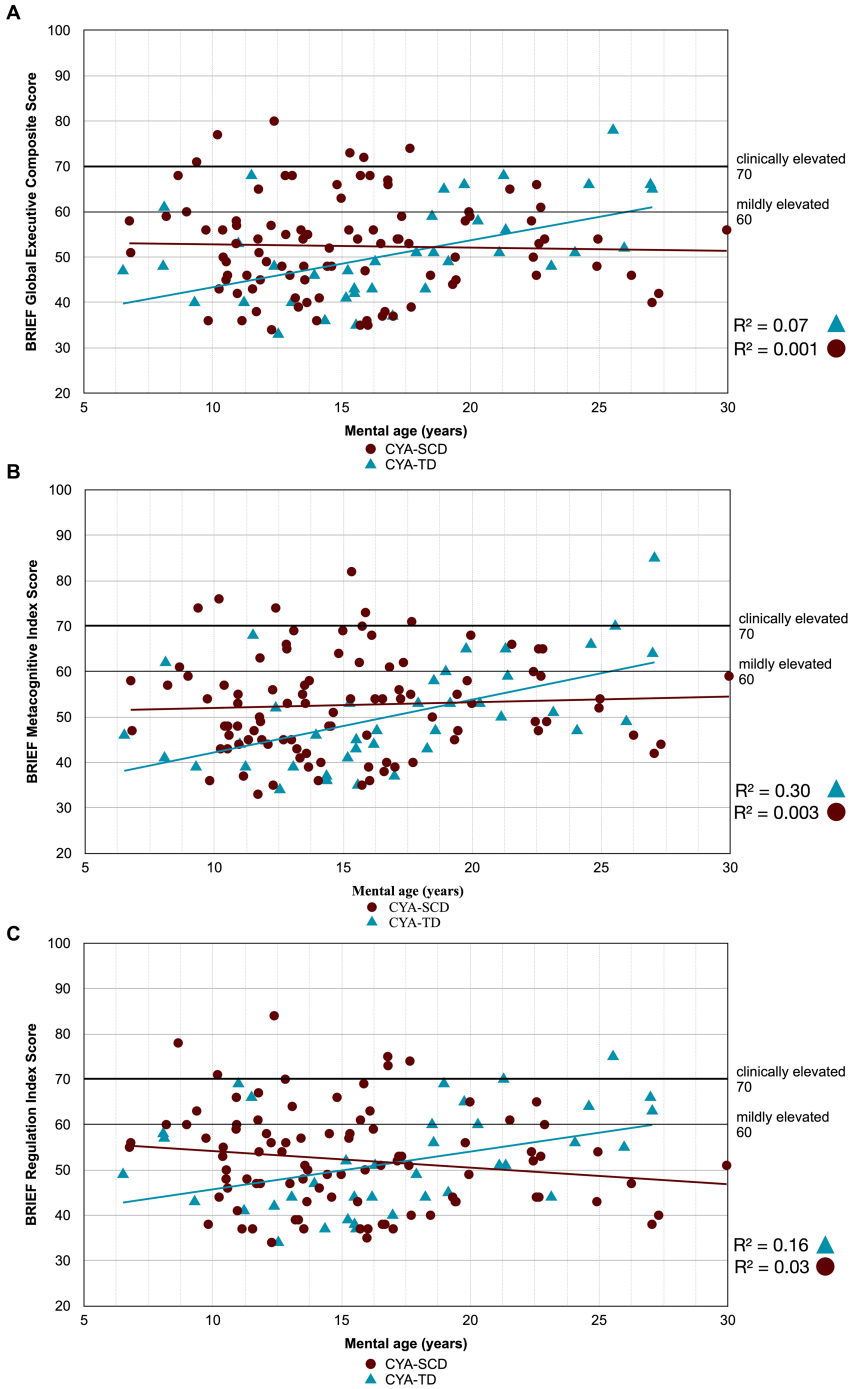
Developmental trajectory of BRIEF Composite Scores. CYA-SCD, children and young adults with sickle cell disease; CYA-TD, children and young adults who are typically developing; BRIEF, Behavior Rating Inventory of Executive Function. **(A)** BRIEF Global Executive Composite Score; **(B)** BRIEF Metacognitive Index Score; **(C)** BRIEF Regulation Index Score.

**Figure 4:**
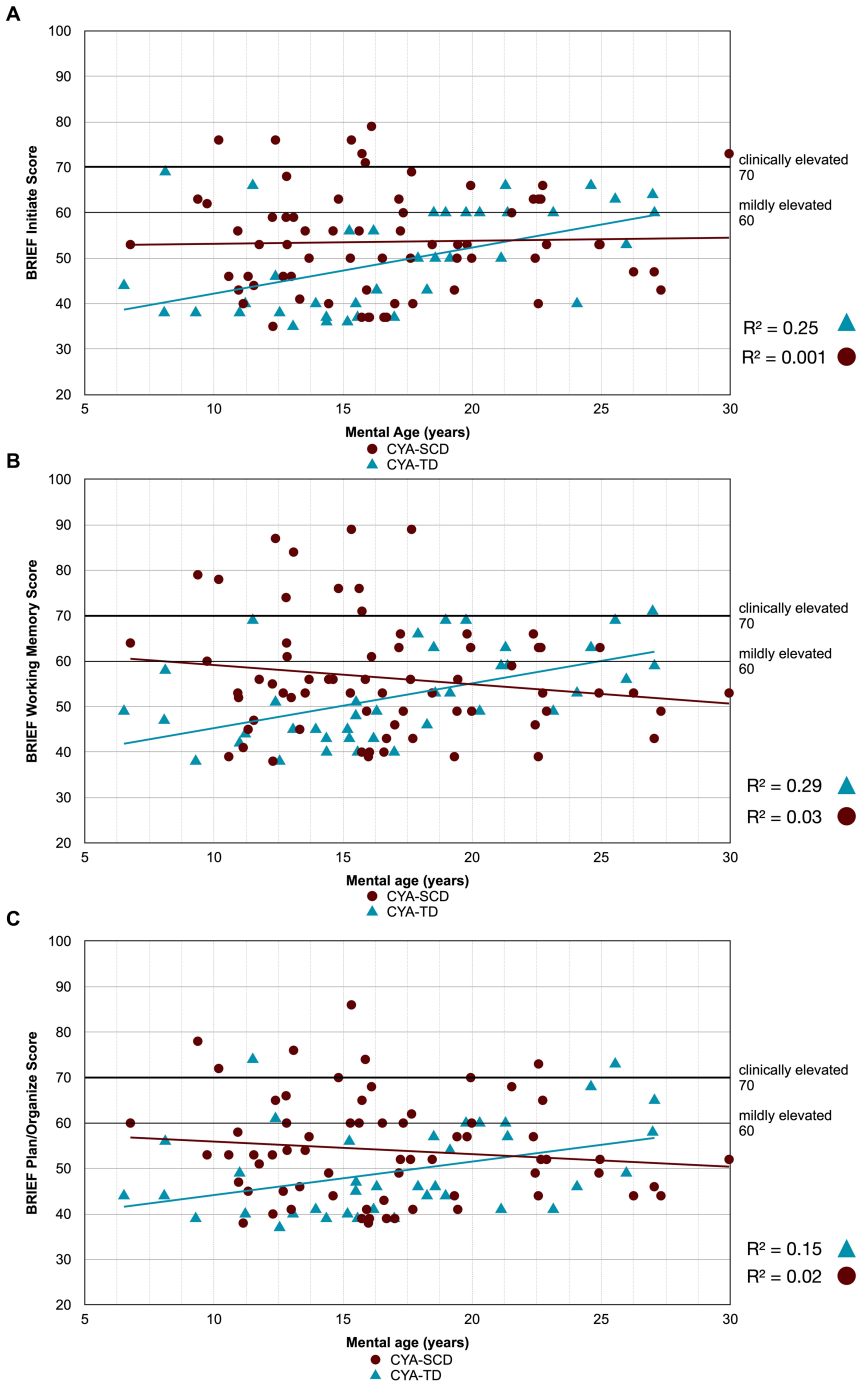
Developmental trajectory of BRIEF MI subscales. CYA-SCD, children and young adults with sickle cell disease; CYA-TD, children and young adults who are typically developing; BRIEF, Behavior Rating Inventory of Executive Function. (A) BRIEF Initiate Score; **(B)** BRIEF Working Memory Score; **(C)** BRIEF Plan/Organize Score.

**Figure 5:**
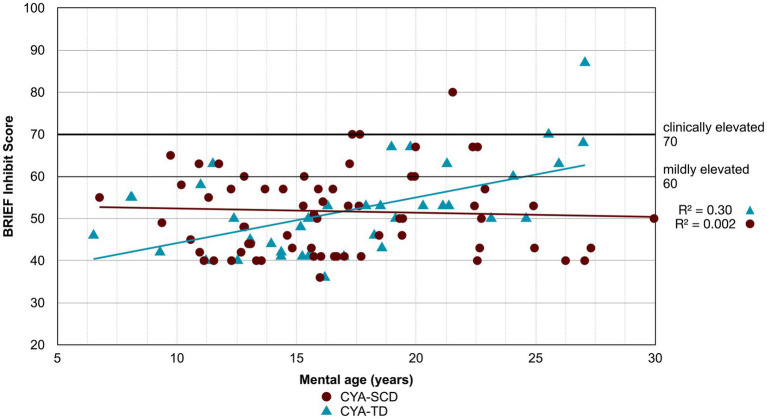
Developmental trajectory of BRIEF BRI Inhibit. CYA-SCD, children and young adults with sickle cell disease; CYA-TD, children and young adults who are typically developing; BRIEF, Behavior Rating Inventory of Executive Function.

**Figure 6:**
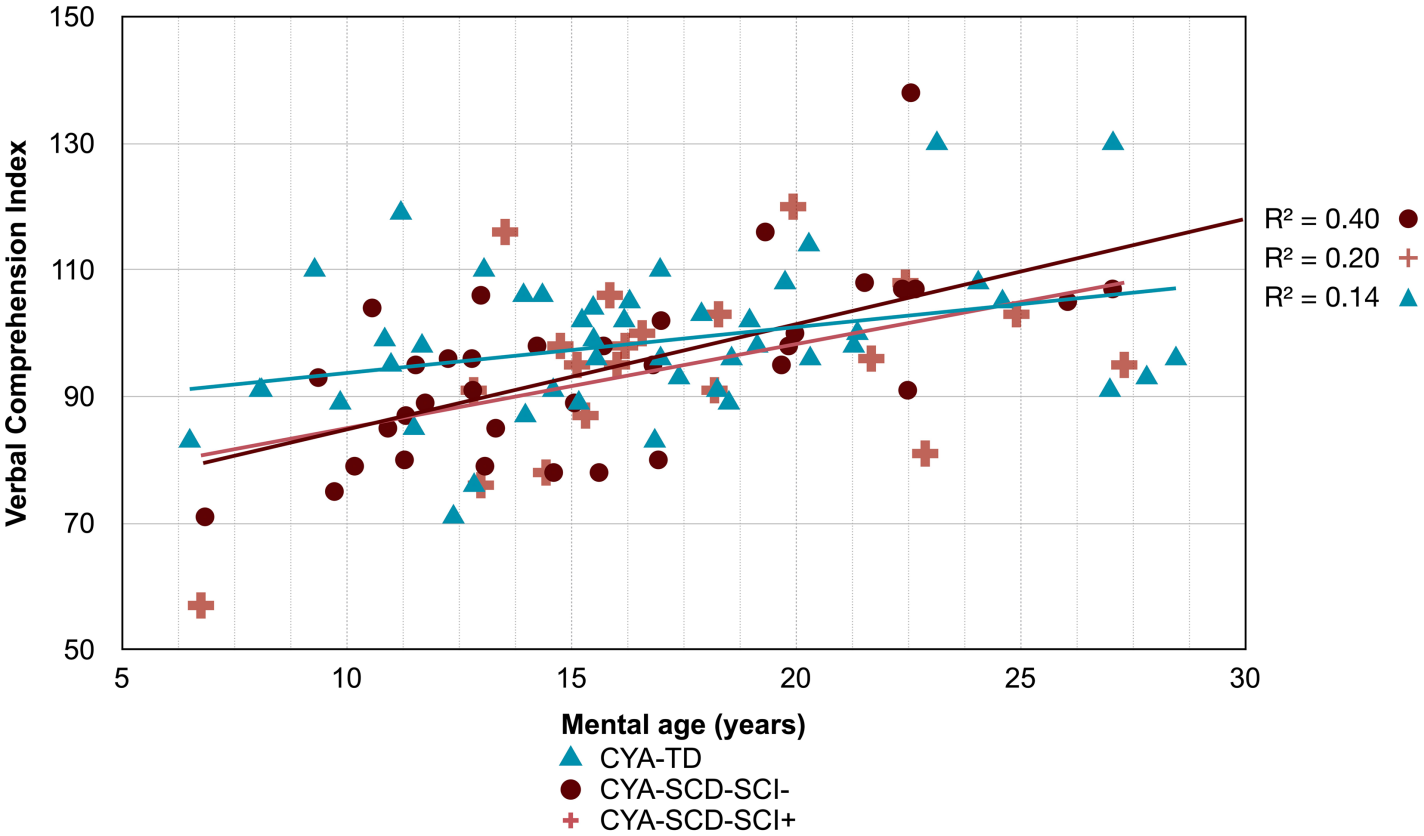
Developmental trajectory of Verbal Comprehension Index. CYA-SCD-SCI+, children and young adults with sickle cell disease with silent cerebral infarcts; CYA-SCD-SCI−, children and young adults with sickle cell disease without silent cerebral infarcts; CYA-TD, children and young adults who are typically developing.

**Figure 7:**
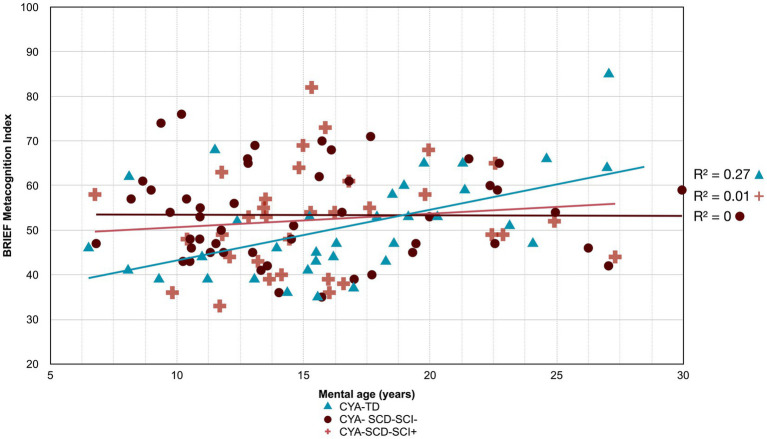
Developmental trajectory of BRIEF MI. CYA-SCD-SCI+, children and young adults with sickle cell disease with silent cerebral infarcts; CYA-SCD-SCI−, children and young adults with sickle cell disease without silent cerebral infarcts; CYA-TD, children and young adults who are typically developing.

As our second step, we examined developmental trajectories between cognition and mental age for CYA-SCD and CYA-TD. ANCOVA models were constructed to evaluate the overall effect of the group in terms of delay at onset and the Group × Mental Age (MA) interaction effect to determine the differences in the rate of cognitive development. Significant results from this analysis are summarised in Table 2 with regressions are plotted in Figures 2–7.

##### 3.3.2.1. Wechsler scales for Intelligence

For Wechsler Indices, there was an overall effect of group for VCI only; thus, the intercepts of the two groups were reliably different at the youngest age of measurement (Table 2 and Figure 2). CYA-SCD appeared to experience a delayed onset in VCI cognitive development, with a lower VCI score of 9.26 points compared to the CYA-TD group at the youngest age. However, there was no significant Group × MA interaction, i.e., verbal comprehension for CYA-SCD did not appear to develop more slowly compared to CYA-TD peers, indicating a possible period for catch up.

##### 3.3.2.2. BRIEF scores

There was an overall effect of group for BRIEF GEC (Figure 3A), BRIEF MI (Figure 3B) and BRIEF MI Initiate, BRIEF MI Working memory and BRIEF MI Plan/Organise subscales (Figures 4A–C), as well as BRIEF BRI (Figure 3C) and BRIEF BRI Inhibit subscale (Figure 5) scores, thus, the intercepts of the CYA-SCD and CYA-TD groups were reliably different at the youngest age of measurement (See Table 2), with CYA-SCD showing worse EF scores at the youngest age. These data indicate that CYA-SCD have a delayed onset for EF compared to CYA-TD, with BRIEF MI Working Memory showing the greatest delay at onset (−27.96) followed by MI Plan/Organise (−21.9) (Table 2 and Figure 4). Significant Group x MA interactions were found for all these measures mentioned, with CYA-SCD demonstrating a slower rate of development compared to their CYA-TD peers, with BRIEF BRI (43%), and MI subscales Working Memory (42%) and Plan/Organise (38%) showing the greatest differences in rate of development.

#### 3.3.3. Rotational analysis (null trajectory analysis)

For cognitive domains where there was a significant relationship between mental age and cognitive scores only for CYA-TD (Supplementary Table S2), but not for the CYA-SCD, an additional rotation analysis or null trajectory analysis was conducted (Supplementary Table S3) to distinguish between Zero trajectory and no systematic relationship. While there were significant relationships between mental age and cognition for the CYA-TD group none were found for CYA-SCD for PRI Block Design and PSI Coding, BRIEF GEC, BRI and its subscale Inhibition, BRIEF MI and its subscales Initiate, Working Memory and Plan/Organize as well as Tower Time score (Supplementary Table S3).

##### 3.3.3.1. No systematic relationship

We found no systematic relationships (i.e., other factors more important than age) for CYA-SCD between age and PSI subscale Coding, PRI subscale Block Design and BRIEF GEC, BRI, MI, and its subscales Working Memory and Plan/Organize, as well as Tower Time score (Supplementary Table S3). The results suggest that there are factors other than disease status contributing to the relationship between age and these cognitive domains. These potential relationships have been explored in the analyses described below (see Sections 3.4 and 3.5).

##### 3.3.3.2. Zero trajectories

We found a significant zero trajectory only for BRIEF subscale Inhibition in relation to mental age in CYA-SCD. Non-significant zero trajectories were observed for the BRIEF GEC and the MI subscale Initiate (Supplementary Table S3). The results suggest that the development of behaviour regulation skills (e.g., inhibition) prematurely plateaued in CYA-SCD.

### 3.4. Cross-sectional developmental trajectories based on SCI

#### 3.4.1. Pre-analysis: simple linear regression

Similar analyses were conducted by dividing the participants in three groups: CYA-SCD and silent cerebral infarction (CYA-SCD-SCI+), CYA-SCD but no SCI (CYA-SCD-SCI−) and CYA-TD. Simple linear regression analyses examining the relationships between mental age and performance on cognition are shown in Supplementary Table S4. As the statistically significant results for the CYA-TD group were for the same domains, we conducted the analyses comparing the CYA-SCD-SCI+ with the CYA-TD group.

#### 3.4.2. Cross-sectional developmental trajectory analysis

##### 3.4.2.1. Weschler scales for Intelligence

Although non-significant, only VCI showed a trend of delay at onset (8 years of age) in the CYA-SCD-SCI+ group with a lower score of 10 points compared to the CYA-TD group at the youngest age (Table 3 and Figure 6). The rate of development did not differ significantly between the three groups.

**Table 3:** Developmental trajectories for CYA-SCD with and without SCI and CYA-TD.

Variable	Slope: cognitive variable = (intercept for group) + (age * gradient)			
	CYA-SCD		CYA-TD	
	SCI+	SCI−		
Wechsler cognitive score				
VCI	80.358 + age * 0.111	78.993 + age * 0.083	90.732 + age * 0.139	
BRIEF				
BRIEF MI	49.600 + age * 0.025	53.513 + age * −0.001	39.734 + age * 0.093	

Developmental trajectories for SCD with and without SCI and controls.												
Variable	Delay at onset SCD		*p*		CI 95%		Rate of development for SCD		*p*		CI 95%	
	CYA-SCD compared with CYA-TD											
	SCI+	SCI−	SCI+	SCI−	SCI+	SCI−	SCI+	SCI	SCI+	SCI−	SCI+	SCI−
Wechsler cognitive score												
VCI	10.37	11.73	0.07	0.85	[67.936, 92.779]	[−16.028, 13.297]	0.79	0.59	0.61	0.36	[−0.150, 0.055]	[−0.080, 0.135]
BRIEF												
BRIEF MI	−9.87	−13.77	0.03	0.11	[41.056, 58.144]	[−21.942, 2.211]	0.26	−0.01	0.05	0.53	[−0.044, 0.095]	[−0.110, 0.057]

Delay at onset = intercept of CYA-TD group – intercept of CYA-SCD group; rate of development = gradient of CYA-SCD group/gradient of CYA-TD group. CYA-SCD-SCI + , children and young adults with sickle cell disease with silent cerebral infarcts; CYA-SCD-SCI−, children and young adults with sickle cell disease without silent cerebral infarcts; CYA-TD, children and young adults who are typically developing; VCI, Verbal Comprehension Index; BRIEF, Behaviour Rating Inventory of Executive Function; BRIEF MI, Metacognition Index.

##### 3.4.2.2. BRIEF scores

The developmental trajectory analysis was significantly different for BRIEF MI, with CYA-SCD showing worse EF scores at the youngest age. At onset, CYA-SCD-SCI+ scored 10 points above CYA-TD, while CYA-SCD-SCI− scored 14 points above CYA-TD, indicative of early EF challenges pertaining to planning and organising, task initiation, self-monitoring, and working memory. The rate of development for BRIEF MI significantly differed between CYA-SCD-SCI+ and CYA-TD which was not the case for the CYA-SCD-SCI− group. CYA-SCD-SCI+ showed a 26% delay in the *rate* of development as compared to the CYA-TD group. This pattern of results suggests that presence of SCI has a significant effect on the development of EF in CYA-SCD (Table 3 and Figure 7).

### 3.5. Cross-sectional developmental trajectory analysis for raw scores

Results for raw scores were tabulated and can be found in the supplementary material for all analyses conducted (see Supplementary Table S5–S8). There was a statistically significant result for the CYA-TD group (Supplementary Table S5) for the Wechsler VCI Similarities and PRI Block design and for the BRIEF GEC, BRI and BRI subtest Inhibit as well as for BRIEF MI subtests Initiate and Organise Material and the Tower Total, while PSI Symbol Search was close to significance. There were no differences in developmental trajectory for the Wechsler raw scores but there were delays at onset and slower rates of development for BRIEF GEC, BRI, BRI Inhibit and MI Initiate raw scores (See Supplementary Table S6). On rotation analysis (see Supplementary Table S7), there was no systematic relationship between BRIEF GEC while there were zero trajectories for BRIEF BRI and BRIEF MI subtest Initiate with a significant delay at onset and a trend for a slower rate of development for CYA-SCD-SCI+ compared with CYA-TD (see Supplementary Table S8).

### 3.6. Exploratory analysis: effect of haemoglobin, CaO_2_, SpO_2_ and SES on cognition

To examine the effect of blood oxygenation measures (haemoglobin, SpO_2_, and CaO_2_) and socioeconomic status (SES) on cognition (scaled scores) in CYA-SCD, we generated simple linear regression models for each of the blood oxygenation measures predicting each cognitive variable separately for CYA-SCD only. All significant results are shown in Table 4 for CYA-SCD and Supplementary Table S9 for CYA-SCD-SCI+.

**Table 4:** Simple linear regression models for blood oxygenation measures and SES predicting cognitive variables in children and young adults with sickle cell disease.

Measure	Cognitive variables		CYA-SCD					
		*N*	*F*	*p*	*R2*	Unst. B	Std. Beta	CI 95%
Haemoglobin								
	VCI	55	5.67	0.02	0.10	0.28	0.31	[0.05, 0.53]
	Similarities	55	4.58	0.04	0.08	0.05	0.28	[0,0.10]
	Vocabulary	106	4.30	0.04	0.04	0.04	0.20	[0, 0.08]
	PSI	106	4.33	0.04	0.04	0.18	0.20	[0.01, 0.36]
	BRIEF Working Memory	54	4.63	0.04	0.08	−0.24	−0.29	[−0.50, −0.02]
CaO_2_								
	VCI	55	7.07	0.01	0.12	2.35	0.34	[0.58, 4.11]
	Similarities	55	5.60	0.02	0.10	0.43	0.31	[0.07, 0.79]
	Vocabulary	106	4.43	0.04	0.04	0.30	0.20	[0.02, 0.59]
	PSI	106	4.65	0.03	0.04	1.38	0.21	[0.11, 2.66]
	Symbol Search	106	4.74	0.03	0.04	0.21	0.06	[0.03, 0.63]
	BRIEF Working Memory	54	4.05	0.05	0.07	−1.72	−0.27	[−3.4, −0.01]
SpO_2_								
	VCI	55	5.18	0.03	0.09	1.47	0.30	[0.17, 2.76]
SES								
	BRIEF Organise Material	64	6.04	0.02	0.07	0	0.30	[0, 0]

CYA-SCD, children and young adults with sickle cell disease; Unst., unstandardised; Std., standardised; CaO_2_, arterial oxygen content; SpO_2_, Oxygen saturation; SES, Socioeconomic status; VCI, Verbal Comprehension Index; PSI, Processing Speed Index; BRIEF, Behaviour Rating Inventory of Executive Function; BRIEF MI, Metacognition Index.

#### 3.6.1. Haemoglobin

For CYA-SCD we found that haemoglobin significantly predicted the variance in VCI (10%, *p* = 0.021), VCI Similarities (8%, *p* = 0.037) and Vocabulary (4% *p* = 0.041), PSI (4%, *p* = 0.04) and BRIEF Working Memory subscale (8.2%, *p* = 0.036). Greater variance was explained by haemoglobin for PRI in the CYA-SCD-SCI+ (29%, *p* = 0.013).

#### 3.6.2. Arterial oxygen content (CaO_2_)

For CYA-SCD CaO_2_ significantly predicted the variance in VCI (12%, *p* = 0.01) and its subscales Similarities (10%, *p* = 0.022) and Vocabulary (4%, *p* = 0.038), PSI (4%, *p* = 0.033) and its subscale Symbol Search (4%, *p* = 0.032) and BRIEF Working Memory (7.2%, *p* = 0.049). Greater variance was explained by CaO_2_ for VCI (22%, *p* = 0.004) and PRI in the CYA-SCD-SCI+ (37%, *p* = 0.005).

#### 3.6.3. Oxygen saturation (SpO_2_)

For CYA-SCD SpO_2_ only significantly predicted the variance in VCI (9%, *p* = 0.027).

#### 3.6.4. Effect of socioeconomic status on cognition

For CYA-SCD, SES significantly predicted the variance in BRIEF MI subscale Organize Material (9%, *p* = 0.017). Greater variance was explained by BRIEF BRI Inhibit in the CYA-SCD-SCI+ (22%, *p* = 0.058), although near-significant.

## 4. Discussion

Compared to intensively researched neurodevelopmental disorders such as Autism and Attention Deficit Hyperactivity Disorder, which are common in the general paediatric population, there are relatively few studies examining cognitive and behavioural profiles in children with underlying medical conditions such as those living with SCD. Developmental delay implies that children do not reach their typical developmental milestone/s in cognition or motor development (48), deviating months from the typically-developing population. A recent systematic review in infants living with SCD (0–48 months) found that 17.5%–50% experience developmental delay in cognition and language development, which had a significant impact by the second year of life (49). Given that developmental delay had received limited attention in CYA-SCD, the main objective of our study was to compare cross-sectional developmental trajectories between CYA-SCD and CYA-TD, who were matched on age and ethnicity. The cross-sectional developmental trajectory approach gives an initial understanding of how cognitive performance might develop with age and differ compared to CYA-TD.

**Figure 8:**
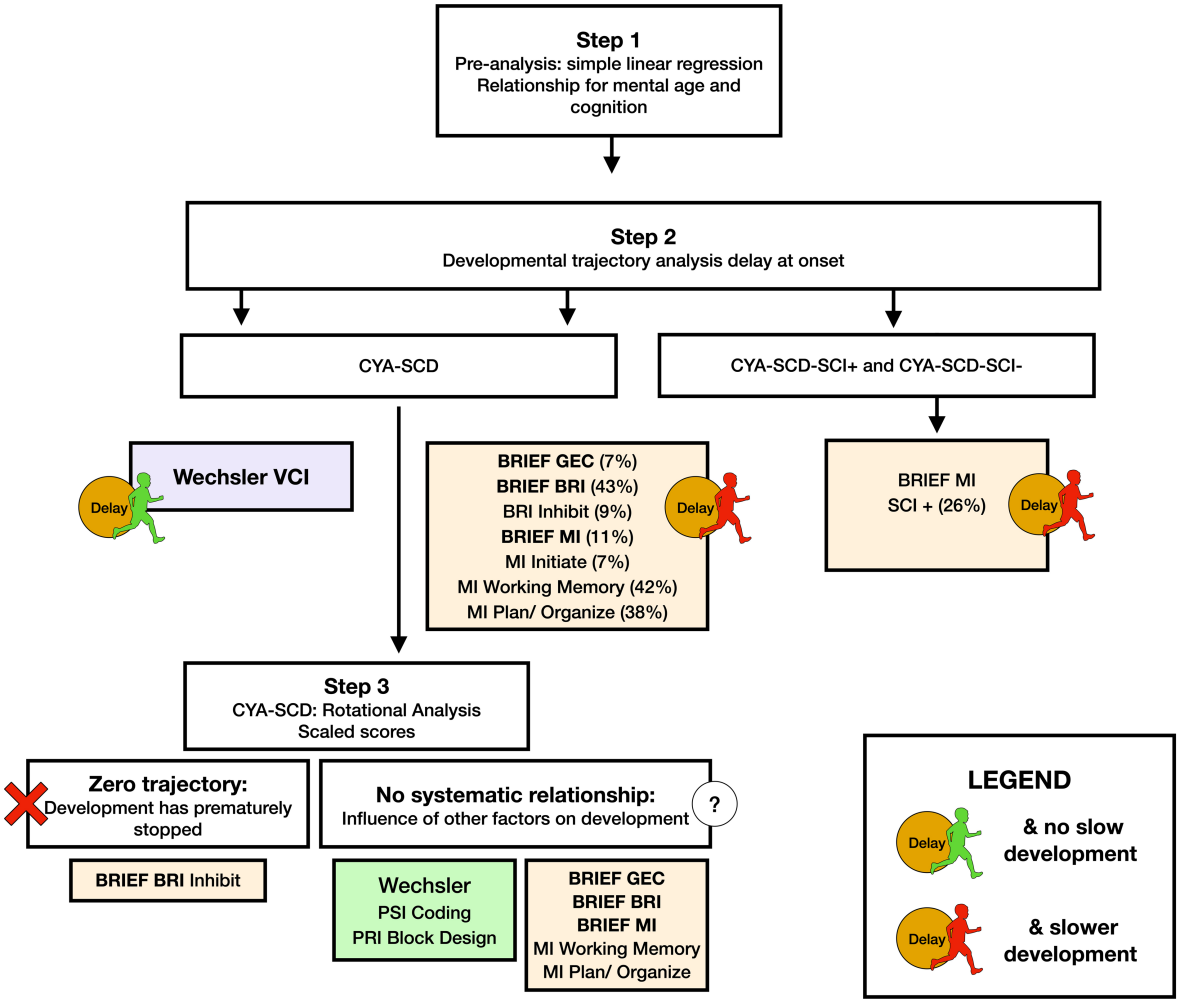
Summary findings of developmental analysis for mental age and cognition in CYA-SCD, and CYA-SCD with and without silent cerebral infarct. CYA-SCD, children and young adults living with sickle cell disease; CYA-SCD-SCI+, children and young adults with sickle cell disease with silent cerebral infarcts; CYA-SCD-SCI−, children and young adults with sickle cell disease without silent cerebral infarcts; BRIEF, Behavior Rating Inventory of Executive Function; BRIEF MI, Metacognitive Index; VCI, Verbal Comprehension Index.

We used common measures of intelligence (Weschler Scales of Intelligence) and EF (BRIEF, D-KEFS). Our findings indicate that CYA-SCD perform poorly on tests of cognitive ability as compared to the CYA-TD. These findings are consistent with previous literature (5, 35, 50). However, the differences are small after controlling for confounders. In our study, CYA-SCD showed developmental delay (difference at age of testing) in verbal comprehension, and developmental delay as well as slower development (rate) in EF (see Summary findings, Figure 8) compared to the CYA-TD. The same was observed for CYA-SCD-SCI+ compared to CYA-TD. However, greater delay and slower development was observed for CYA-SCD-SCI+ and EF (BRIEF MI: meta cognition), suggesting greater EF challenges in CYA-SCD-SCI+ (see Summary findings, Figure 8).

**Figure 9:**
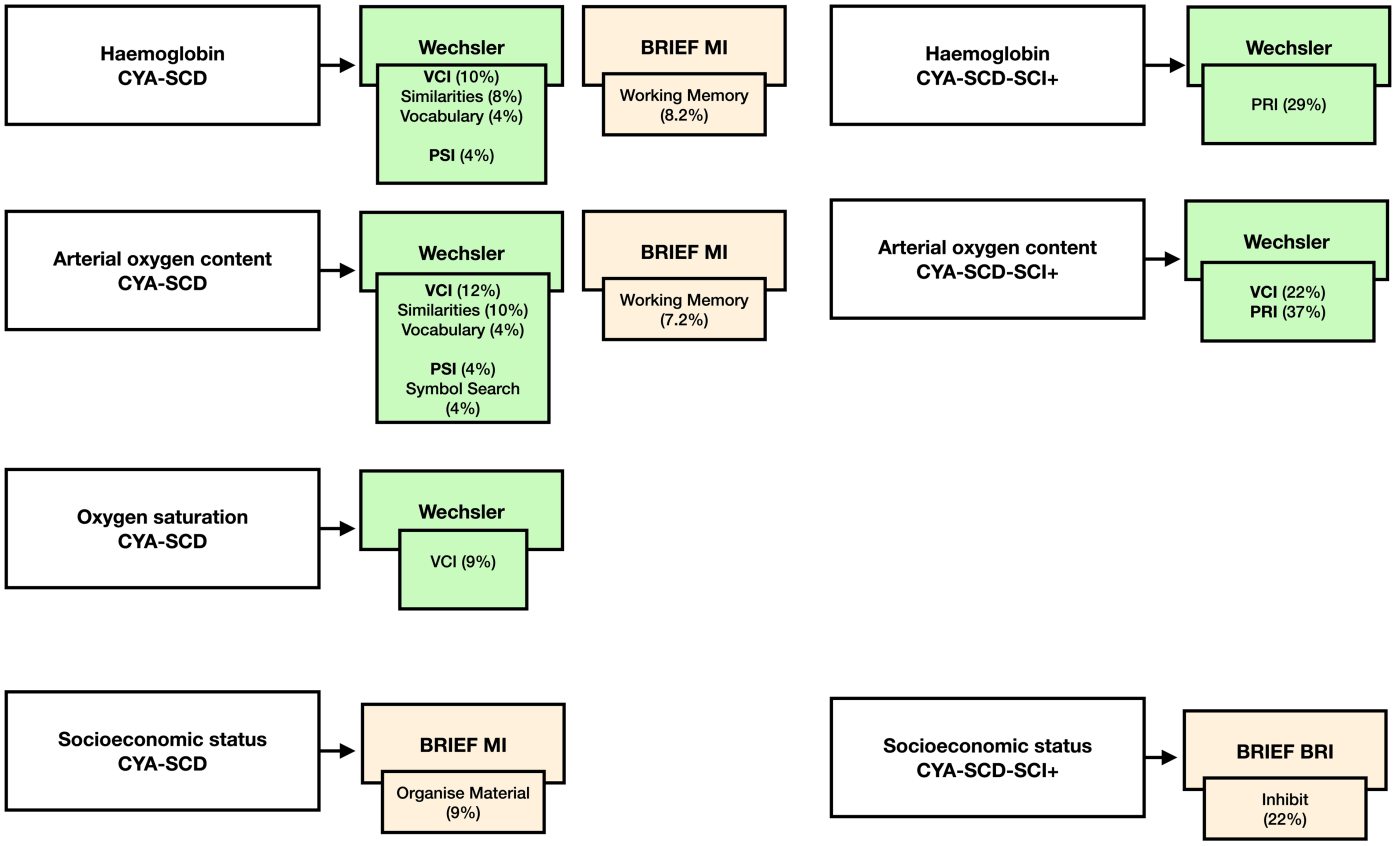
Summary findings of the effect of haemoglobin, CaO_2_, SpO_2_ and SES on cognition CYA-SCD and CYA-SCD-SCI+. CYA-SCD-SCI+, children and young adults with sickle cell disease with silent cerebral infarcts; CYA-SCD-SCI−, children and young adults with sickle cell disease without silent cerebral infarcts; BRIEF, Behavior Rating Inventory of Executive Function; VCI, Verbal Comprehension Index; PSI, Processing Speed Index; RRI, Perceptual Reasoning Index.

Our initial analyses prompted us to examine if blood oxygenation measures such as haemoglobin, SpO_2_, and CaO_2_ have a significant effect on cognitive development in CYA-SCD (see Summary findings, Figure 9). We found that all blood oxygenation measures (haemoglobin, SpO_2_, and CaO_2_) had a significant relationship with verbal comprehension, whereas haemoglobin and CaO_2_ alone predicted processing speed, suggesting a dominant effect of haemoglobin in this analysis. Interestingly, haemoglobin and socioeconomic status (SES) alone predicted caregiver and self-reported EF. In CYA-SCD-SCI+, haemoglobin and CaO_2_ predicted perceptual reasoning (problem solving), while SES alone predicted caregiver and self-reported EF. These data suggest that development of cognitive function, especially EF and problem solving skills, are impaired in CYA-SCD and may be associated with poor blood oxygenation, possibly leading to haemodynamic stress, during development (51, 52).

### 4.1. Cross-sectional developmental trajectories

#### 4.1.1. Wechsler scales

The main findings of our paper indicate that CYA-SCD tend to have a delay in verbal comprehension skills, but they also appear to have a catch-up period and are no longer delayed as young adults. However, this initial delay could impact on later successful reading skills (50) and successful academic performance in school-aged children (53). Additionally, research in healthy infants between the ages of 7–12 months has shown a significant correlation between language development (Mullen Scales of Early Learning) and grey and white matter volume (e.g., subcortical region hippocampus) (54). Specifically, for CYA-SCD, Steen et al. (55) demonstrated that grey matter volume growth is delayed in children living with SCD aged 3.9–18.5 years. Another factor to consider is that grey matter volume reduces between 2 to 9 years of age in parallel with white matter myelination, after which grey matter volumes stabilise until late adulthood (55). Therefore, the observed developmental delay in verbal comprehension could be related to delayed grey matter volume development in CYA-SCD. Neuroimaging studies have shown associations between frontal lobe volumes and verbal comprehension tasks in CYA-TD (56). While this association remains unexplored in CYA-SCD, it is possible that reduced volumes in the frontal lobe could be contributing factors to the delay observed in verbal comprehension development.

Additional research in CYA-SCD aged 7–17 years found lower verbal comprehension performance (Woodcock-Johnson-III) in those with greater frontal–parietal cortical lesions (57), which was also related to lower cognitive control (Examiner Battery: measuring inhibition and dysregulation of EF). Interestingly, cognitive control is important for successful language comprehension (58). We know that children living with SCD experience EF difficulties (59) affecting the central executive and working memory performance. It is possible that there is a relationship between EF and verbal comprehension in CYA living with SCD as well, as previously shown in a healthy adult population (60). However, these hypotheses need further investigation.

It is important to mention that there may be other factors contributing to the delay observed in verbal comprehension, such as prematurity (61), SES (62), low school attendance and missed schooling due to hospital visits and health related problems. For example, painful crisis is common and is also associated with reduced grey matter volumes in CYA-SCD (63). This evidence suggests that there could be multiple factors affecting verbal comprehension development in CYA-SCD which need to be explored in future research.

#### 4.1.2. Executive function

Several studies have documented impaired EF skills in CYA-SCD. Our results not only agree with the previous literature (64, 65), but demonstrate that the development of EF skills in CYA-SCD are not only delayed, but show a slower rate of development. Our findings indicate that EF difficulties continue into young adulthood. Most difficulties seem to relate to the child’s ability to control and regulate emotions and behaviour, important for self-regulation (measured on the BRIEF BRI). Our research is the first to show that CYA-SCD plateau prematurely on the development impulsivity and inhibitory control (measured on the BRIEF BRI Inhibition). Recent research in children living with SCD aged 8–15 years found that impulsivity (Conners’ Continuous Performance Test) was negatively associated with health-related quality of life (Paediatric Quality of Life Inventory Sickle Cell Disease Module) (66). There is also a high incidence of ADHD diagnosis (25%) in children living with SCD aged 8–16 years, which may contribute to EF difficulties in these patients (67).

Parents of children living SCD observe behavioural difficulties already at a young age. We found that they have difficulties anticipating an activity or task, develop ideas and problem-solving strategies, which are crucial for efficient time management and planning (measured on the BRIEF MI). Similar findings were observed in 8- to 12-year old children living with SCD compared to typically developing controls (68). Berg et al. (68) found higher scores on the parent and teacher BRIEF for MI and GEC. The authors mention that parents of children living with SCD observe EF difficulties in daily behaviour such as organizing and remembering things (i.e., homework materials, daily chores) and initiating behaviour to start tasks.

Interestingly, older CYA-TD in our sample had more EF difficulties compared to CYA-SCD. There are multiple factors which need to be considered when understanding EF development. In addition to changes in adolescence which may be prolonged into early adult life, the impact of psychosocial factors (i.e., family environment, parental support and engagement) (69) and even school environment (70) could contribute to individual differences in EF-skills development. Studies have shown that family function plays an important role in the development of EF skills early in life (71). Downes et al. (71) noted that a positive family environment and functioning significantly impacted attention control and cognitive flexibility in pre-schoolers living with SCD. It is likely that these functions, especially attention control, may have a cascading effect on the development of other EF skills, suggesting that efforts to support families during the early years could benefit EF development later in life. However, health plays a crucial role in children with SCD, which needs to be considered as well. Current research in people living with SCD in a similar age range to our study (6–31 years) has shown that this group is vulnerable to EF deficits because of cerebral haemodynamic stress related to chronic anaemia and oxygen desaturation (52). For example, cerebral blood flow increases to maintain adequate oxygen delivery to the cortex and is an indicator of cerebral hemodynamic stress, which is associated with worse EF, as measured by the D-KEFS Tower Test (72). One neuroimaging study in the SCD population showed reduced resting state activity in the default mode network (important for cognitive processing) but increased activity in the pain processing regions suggesting allocation of cognitive resources towards pain management which may hinder normal EF development (73).

Besides multiple bio-psychosocial factors that affect EF development, it is also possible that our results could be attributed to recruitment methods. Other cohort participants have attended a neuropsychological evaluation based on a medical referral for academic concerns, whereas we recruited participants from clinics and community settings regardless of any history of cognitive difficulties, so it is probable that our sample has a broader range of EF profiles, making our results applicable in a wider clinical context.

#### 4.1.3. Presence of SCI and cognition

We found that CYA-SCD-SCI+ showed more delay at onset and a slower rate of development for BRIEF MI. These results are in line with previous literature showing an association between poor EF and SCI status (74). Although we did not examine the site of SCI lesions, previous studies looking at SCI presence have noted that frontal lobe SCI lesions could explain poor EF, especially metacognition, such as inhibition and working memory (74, 75). People living with SCD and SCI tend to have poorer white matter microstructural integrity as compared to people living with SCD without SCI which may affect EF as well (76). However, more diffusion tensor-based imaging studies as well as functional connectivity on MRI studies are needed to explore the multidimensional relationship between structural connectivity and EF in detail.

#### 4.1.4. Factors affecting cognition

In addition to finding that verbal comprehension was delayed in participants with SCI (although not significantly), we also found that haemoglobin, SpO_2_, and CaO_2_ are all predictors of verbal comprehension in CYA-SCD. Haemoglobin and CaO_2_ were also associated with processing speed. These results are in line with previous studies exploring effects of blood oxygenation measures on cognition in people living with SCD. Using a path-analysis model, Hogan et al. (34) found that reduced SpO_2_ is compensated by increased cerebral blood flow to the brain which results in lowered verbal IQ in adolescents living with SCD (mean age 17.4 years). Several studies of blood oxygenation measures and cognition have found that reduced haemoglobin is associated with reduced IQ in adolescents and young adults (51) and also neurodevelopmental delay in 9- to 12-month old infants living with SCD (77). Similarly, reduced processing speed was linked to lower CaO_2_, poor white matter microstructural integrity in (35) and reduced white matter structural connectivity (78, 79) in CYA-SCD. Taken together, these data suggest that cerebral haemodynamic stress, central to sickle cell anaemia pathology, may result in poor long-term cognitive outcomes.

### 4.2. Limitations

There were some limitations to our study, which should be considered for future research. We did not include other factors that have been shown to impact on cognition in people living with SCD, such as: sleep and cortisol (80) and medical interventions (i.e., hydroxyurea and blood transfusions) (81, 82), and cortical brain areas affected by stroke. We did not exclude CYA-TD with SCI. The cross-sectional developmental trajectory approach gives only an initial understanding as to how cognitive performance might develop with age but needs to be validated in longitudinal follow-up studies. Therefore, observing these children from birth, taking both disease-related (83), social and environmental factors (84) into account would be the ideal way to assess developmental changes and to control for potential confounding variables. However, this methodology is challenging due to funding restrictions and participants being lost to follow-up. Although the Wechsler tests are widely used they may underestimate the influence of general and fluid intelligence on the other cognitive domains (85). Various subtests of the Wechsler tests require intact EF although it is not measured directly, such as the subtest digit span backwards included in the Working Memory Index (86). We can find EF in most cognitive processing. For example, research in young and older adults (18–88 years) has shown that FSIQ, Verbal Comprehension Index and Working Memory Index on the WAIS-IV were strongly correlated with EF skills measured on the Neuropsychological Assessment Battery, suggesting an impact of EF on other cognitive domains (87). Future research should carefully select quantitative cognitive assessments, such as the NIH Toolbox assessing EF in CYA living with sickle cell disease (23, 88). Adolescents and young adults living with SCD and executive dysfunction may also have learning difficulties (89), which we did not screen for, although the participants in our cohorts had FSIQ within the average range and only 4 (4.4%) showed an FSIQ ≤69. Current research addresses this issue and recommends screening for neurodevelopmental disorders (e.g., autism) to implement early interventions, which can result in better treatment efficacy (90). Screening should also consider language, education, and culture bias, since most of the neuropsychological assessments are not language and culture free. Further, the BRIEF is a caregiver report which potentially makes it more vulnerable to bias. Future research could also incorporate a mixed method approach (i.e., using qualitative and quantitative measures). Teachers and caregiver perspective on cognitive abilities would provide some needed background to the current findings.

### 4.3. Conclusion

Our findings demonstrate that when considering cognitive developmental trajectories in CYA-SCD, their cognitive profiles are not impaired, but verbal comprehension is delayed with later catch-up and EF appears to develop at a slower rate. It is recommended that, in addition to initiation of medical treatment, early cognitive intervention and educational support could strengthen cognitive development.

## Data availability statement

The original contributions presented in the study are included in the article/Supplementary material, further inquiries can be directed to the corresponding authors.

## Ethics statement

The studies involving human participants were reviewed and approved by West London NHS (SAC; 05/Q0408/42, 11/EM/0084, 15/LO/0347), Yorkshire NHS (POMS; 15/YH/0213), and University College London (14475/001). Written informed consent to participate in this study was provided by the participants’ legal guardian/next of kin.

## Author contributions

MK conceptualised the paper, completed the review, and drafted the manuscript. MK and HS were involved in data collection. MK and SH analysed the data supervised by FK, AH, and DD. HS and AH contributed to the review and provided feedback on the manuscript. FK and DD conceptualised the paper and provided feedback on the manuscript. All authors contributed to the article and approved the submitted version.

## Funding

MK and HS were funded by Action Medical Research (GN2509).

## Conflict of interest

The authors declare that the research was conducted in the absence of any commercial or financial relationships that could be construed as a potential conflict of interest.

## Publisher’s note

All claims expressed in this article are solely those of the authors and do not necessarily represent those of their affiliated organizations, or those of the publisher, the editors and the reviewers. Any product that may be evaluated in this article, or claim that may be made by its manufacturer, is not guaranteed or endorsed by the publisher.

## Figures and Tables

**Table:** 

BRIEF	Behavior Rating Inventory of Executive Function
BRIEF GEC	BRIEF Global Executive Composite
BRIEF MI	Metacognitive Index
BRIEF RI	Regulation Index
CaO_2_	Arterial oxygen content
CYA-SCD	children and young adults living with SCD
EF	Executive function
FSIQ	Full Scale IQ score
Hb	Haemoglobin
MA	Mental age
MRT	Magnetic resonance imaging
PSI	Processing Speed Index
PRI	Perceptual Reasoning Index
SCA	Sickle cell anaemia
SCD	Sickle cell disease
SCI	Silent cerebral infarction
SES	Socioeconomic status
SpO_2_	Oxygen saturation
CYA-TD	Typically developing children and young adults
VCI	Verbal Comprehension Index
WMI	Working Memory Index

## Footnotes

^1^http://imd-by-postcode.opendatacommunities.org/imd/2019
