# Supplementary material for: Mind the gap: trajectory of cognitive development in young individuals with sickle cell disease: a cross-sectional study
Source: Front Neurol. 2023 Jul 25;14:1087054. doi: 10.3389/fneur.2023.1087054 (PMC10408298; doi:10.3389/fneur.2023.1087054)
Supplement: Supplementary file 1 [file Data_Sheet_1.docx]

| **Supplement Table S1**  *Participant comparison for children and young adults living with sickle cell disease with or without silent cerebral infarcts and for typically developing children and young adults* | | | | | | | | | | | | | | | |  |
| --- | --- | --- | --- | --- | --- | --- | --- | --- | --- | --- | --- | --- | --- | --- | --- | --- |
|  | | | | | | | | |  |  |  |  |  |  |  |  |
|  | **CYA-SCD** | | | | | | ***CYA-TD*** | | | | ***Group comparison*** | | ***CYA-SCD-SCI+ compared to CYA-TD*** | | ***CYA-SCD-SCI+ compared to CYA-SCD-SCI-*** | |
|  |  |  |  |  |  |  |  |  |  |  |  |  |  |  |  |  |
|  | ***N*** | **SCI+** | ***CI 95%*** | ***N*** | **SCI-** | ***CI 95%*** | ***N*** |  | | ***CI 95%*** | ***p^1^*** | ***Effect size*** | ***p^2^*** | ***Effect size*** | ***p^3^*** | ***Effect size*** |
| **Demographics** |  |  |  |  |  |  |  |  | |  |  |  |  |  |  |  |
| Gender | 42 | 22 Male |  | 64 | 32 Male |  | 46 | 19 Male | |  |  |  |  |  |  |  |
|  |  | 20 Female |  |  | 32 Female |  |  | 27 Female | |  |  |  |  |  |  |  |
|  |  |  |  |  |  |  |  |  | |  |  |  |  |  |  |  |
| Age in years | 42 | 17.21 (4.62) | [15.77,  18.65] | 64 | 16.61 (5.18) | [15.31,  17.90] | 46 | 17.09 (5.09) | | [15.58,  18.60] |  |  |  |  | 0.05* | 0.14 |
|  |  | 8.54-27.90 |  |  | 8.02-29.40 |  |  | 8.24-30.67 | |  |  |  |  |  |  |  |
|  |  |  |  |  |  |  |  |  | |  |  |  |  |  |  |  |
| SES | 40 | 8843.80 (5742.28) | [7007.33,  10680.27] | 61 | 10540.80 (6681.26) | [8829.65,  12251.95] | 46 | 9289.48 (6468.03) | | [7368.71,  11210.25] |  |  |  |  | 0.05* | 0.27 |
|  |  | 1443-32371 |  |  | 3153-31444 |  |  | 3056-32371 | |  |  |  |  |  |  |  |
|  |  |  |  |  |  |  |  |  | |  |  |  |  |  |  |  |
| SpO_2_ % | 42 | 95.79 (3.39) | [94.73,  96.84] | 64 | 97.25 (2.04) | [96.74,  97.76] | 46 | 98.59 (1.33) | | [98.19,  98.98] | 0.001 | 0.17 | <.001 | 0.24 | 0.21 | 0.04 |
|  |  | 89-100 |  |  | 92-100 |  |  | 93-100 | |  |  |  |  |  |  |  |
|  |  |  |  |  |  |  |  |  | |  |  |  |  |  |  |  |
| CaO_2_ mL/d | 42 | 11.5 (1.99) | [10.88,  12.12] | 64 | 11.87 (1.89) | [11.40,  12.35] | 46 | 18.12 (1.05) | | [17.81,  18.43] | <.001 | 0.77 | <.001 | 0.85 | 0.42 | 0.01 |
|  |  | 8.20-16.66 |  |  | 7.79-17.55 |  |  | 16.85-20.68 | |  |  |  |  |  |  |  |
|  |  |  |  |  |  |  |  |  | |  |  |  |  |  |  |  |
| Hemoglobin g/L | 42 | 87.03 (14.33) | [82.57,  91.50] | 64 | 88.71 (14.24) | [85.15,  92.26] | 46 | 134.89 (8.65) | | [132.32,  137.46] | <.001 | 0.76 | <.001 | 0.84 | 0.62 | 0.00 |
|  |  | 64-130 |  |  | 60-134 |  |  | 126-152 | |  |  |  |  |  |  |  |
| Hematocrit % | 42 | 25.87 (4.61) | [24.43,  27.30] | 64 | 26.65 (4.56) | [25.51,  27.79] | 46 | 39.58 (5.98) | | [37.80,  41.35] | <.001 | 0.61 | <.001 | 0.65 | 0.48 | 0.01 |
|  |  | 18.40-39.20 |  |  | 16.10-40 |  |  | 3.4-45.3 | |  |  |  |  |  |  |  |
| **Wechsler Cognitive Scores** | | | | | | | | | | | | | | | | |
| **FSIQ** | 42 | 93.07 (13.16) | [89.92,  96.49] | 64 | 93.20 (13.16) | [89.91,  96.49] | 46 | 97.52 (12.36) | | [93.85,  101.19] | 0.30 | 0.02 | 0.21 | 0.02 | 0.91 | 0.00 |
|  |  | 64-122 |  |  | 64-122 |  |  | 75-130 | |  |  |  |  |  |  |  |
|  |  |  |  |  |  |  |  |  | |  |  |  |  |  |  |  |
| **VCI** | 20 | 94.79 (14.35) | [87.98,  101.41] | 35 | 94.31 (13.47) | [89.69,  98.94] | 46 | 98.57 (11.76) | | [95.07,  102.06] | 0.32 | 0.03 | 0.35 | 0.02 | 0.81 | 0.00 |
|  |  | 57-120 |  |  | 71-138 |  |  | 71-130 | |  |  |  |  |  |  |  |
|  |  |  |  |  |  |  |  |  | |  |  |  |  |  |  |  |
| Similarities | 20 | 8.80 (2.98) | [7.40,  10.20] | 35 | 8.77 (2.68) | [7.85,  9.69] | 46 | 10.09 (2.25) | | [9.42-  10.75] | 0.07 | 0.06 | 0.11 | 0.04 | 0.89 | 0.00 |
|  |  | 3-17 |  |  | 4-15 |  |  | 6-16 | |  |  |  |  |  |  |  |
|  |  |  |  |  |  |  |  |  | |  |  |  |  |  |  |  |
| Vocabulary | 42 | 9.07 (3.01) | [8.44,  9.94] | 64 | 9.19 (3.01) | [8.43,  9.94] | 46 | 9.96 (2.77) | | [9.13,  10.78] | 0.38 | 0.01 | 0.26 | 0.02 | 0.98 | 0.00 |
|  |  | 2-17 |  |  | 2-17 |  |  | 5-17 | |  |  |  |  |  |  |  |
|  |  |  |  |  |  |  |  |  | |  |  |  |  |  |  |  |
| **PRI** | 20 | 90 (9.27) | [85.66,  94.34] | 35 | 90.57 (12.04) | [86.44,  94.71] | 46 | 97.04 (10.39) | | [93.96,  100.13] | 0.03 | 0.08 | 0.02 | 0.09 | 0.56 | 0.01 |
|  |  | 77-110 |  |  | 61-111 |  |  | 73-117 | |  |  |  |  |  |  |  |
|  |  |  |  |  |  |  |  |  | |  |  |  |  |  |  |  |
| Block Design | 20 | 7.10 (2.45) | [5.95,8.24] | 35 | 7.51 (2.06) | [6.81,  8.22] | 46 | 8.48 (2.20) | | [7.83,  9.13] | 0.10 | 0.05 | 0.04 | 0.07 | 0.58 | 0.01 |
|  |  | 3-14 |  |  | 3-12 |  |  | 4-13 | |  |  |  |  |  |  |  |
|  |  |  |  |  |  |  |  |  | |  |  |  |  |  |  |  |
| Matrix Reasoning | 42 | 9.81 (2.91) | [8.90,  10.72] | 64 | 9.44 (2.91) | [8.71,  10.16] | 46 | 10.70 (2.56) | | [9.93,  11.46] | 0.09 | 0.03 | 0.13 | 0.03 | 0.66 | 0.00 |
|  |  | 1-15 |  |  | 2-16 |  |  | 4-15 | |  |  |  |  |  |  |  |
|  |  |  |  |  |  |  |  |  | |  |  |  |  |  |  |  |
| **WMI** | 42 | 91.95 (13.43) | [87.77,  96.14] | 64 | 92.47 (14.65) | [88.81,  96.13] | 46 | 99.13 (13.28) | | [95.19,  103.07] | 0.03 | 0.05 | 0.01 | 0.08 | 0.67 | 0.00 |
|  |  | 62-126 |  |  | 56-136 |  |  | 65-136 | |  |  |  |  |  |  |  |
|  |  |  |  |  |  |  |  |  | |  |  |  |  |  |  |  |
| Digit Span | 42 | 8.90 (2.87) | [8.01,  9.80] | 64 | 8.72 (2.51) | [8.10,  9.35] | 46 | 10.30 (2.71) | | [9.59,  11.11] | 0.01 | 0.06 | 0.01 | 0.08 | 0.98 | 0.00 |
|  |  | 4-2017 |  |  | 3-14 |  |  | 5-19 | |  |  |  |  |  |  |  |
|  |  |  |  |  |  |  |  |  | |  |  |  |  |  |  |  |
| Arithmetic | 40 | 8.73 (2.88) | [7.80,  9.65] | 60 | 8.18 (2.75) | [7.37,  9.00] | 42 | 9.86 (2.75) | | [9.00,  10.71] | 0.04 | 0.05 | 0.09 | 0.04 | 0.40 | 0.01 |
|  |  | 4-15 |  |  | 5-18 |  |  | 5-18 | |  |  |  |  |  |  |  |
|  |  |  |  |  |  |  |  |  | |  |  |  |  |  |  |  |
| **PSI** | 42 | 88.77 (12.07) | [85.00,  92.52] | 64 | 89.45 (13.54) | [86.07,  92.84] | 46 | 97.74 (14.93) | | [93.31,  102.17] | 0.01 | 0.07 | 0.01 | 0.08 | 0.99 | 0.00 |
|  |  | 65-115 |  |  | 59-118 |  |  | 53-122 | |  |  |  |  |  |  |  |
|  |  |  |  |  |  |  |  |  | |  |  |  |  |  |  |  |
| Coding | 42 | 8.10 (2.22) | [7.40,  8.79] | 64 | 7.5 (2.62) | [6.85,  8.15] | 46 | 9.35 (3.08) | | [8.43,  10.26] | 0.01 | 0.07 | 0.10 | 0.03 | 0.21 | 0.02 |
|  |  | 3-12 |  |  | 2-13 |  |  | 2-15 | |  |  |  |  |  |  |  |
|  |  |  |  |  |  |  |  |  | |  |  |  |  |  |  |  |
| Symbol Search | 42 | 7.86 (3.11) | [6.89,  8.83] | 64 | 8.75 (2.94) | [8.02,  9.48] | 46 | 9.50 (2.86) | | [8.65,  10.35] | 0.10 | 0.03 | 0.03 | 0.05 | 0.25 | 0.01 |
|  |  | 1-14 |  |  | 1-15 |  |  | 1-15 | |  |  |  |  |  |  |  |
| Cancellation | 42 | 8.67 (3.51) | [7.57,  9.76] | 64 | 10.20 (3.51) | [9.32,  11.08] | 45 | 10.31 (2.98) | | [9.42,  11.21] | 0.05 | 0.04 | 0.02 | 0.07 | 0.08 | 0.03 |
|  |  | 2-2018 |  |  | 2-19 |  |  | 3-18 | |  |  |  |  |  |  |  |
| **Executive Function Scores** | | | | | | | | | | | | | | | | |
| **BRIEF GEC** | 33 | 52.18 (10.79) | [48.36,  56.00] | 49 | 53.41 (10.53) | [50.38,  56.43] | 34 | 50.76 (10.14) | | [47.23,  54.30] | 0.44 | 0.02 | 0.57 | 0.01 | 0.67 | 0.00 |
|  |  | 35-73 |  |  | 35-77 |  |  | 35-68 | |  |  |  |  |  |  |  |
|  |  |  |  |  |  |  |  |  | |  |  |  |  |  |  |  |
| **BRIEF BRI** | 33 | 51.76 (10.32) | [48.10,  55.42] | 49 | 53.20 (10.80) | [50.10,  56.30] | 34 | 51.76 (10.48) | | [48.11,  55.42] | 0.69 | 0.01 | 0.93 | 0.00 | 0.63 | 0.00 |
|  |  | 35-75 |  |  | 37-78 |  |  | 37-70 | |  |  |  |  |  |  |  |
|  |  |  |  |  |  |  |  |  | |  |  |  |  |  |  |  |
| Inhibit | 19 | 51.05 (8.46) | [46.98,  55.13] | 35 | 51.29 (10.43) | [47.70,  54.87] | 34 | 51.59 (10.55) | | [47.91,  55.27] | 0.58 | 0.01 | 0.16 | 0.04 | 0.56 | 0.01 |
|  |  | 36-67 |  |  | 40-80 |  |  | 36-87 | |  |  |  |  |  |  |  |
|  |  |  |  |  |  |  |  |  | |  |  |  |  |  |  |  |
| Shift | 19 | 52.68 (10.76) | [47.50,  57.87] | 35 | 54.54 (11.85) | [50.47,  58.61] | 34 | 53.26 (10.64) | | [49.55,  56.98] | 0.86 | 0.00 | 0.65 | 0.00 | 0.87 | 0.00 |
|  |  | 38-69 |  |  | 38-81 |  |  | 38-73 | |  |  |  |  |  |  |  |
|  |  |  |  |  |  |  |  |  | |  |  |  |  |  |  |  |
| Emotional Control | 19 | 53.84 (13.72) | [47.23,  60.46] | 34 | 50.82 (11.17) | [46.92,  54.72] | 34 | 51.79 (12.69) | | [47.40,  56.19] | 0.74 | 0.01 | 0.92 | 0.00 | 0.33 | 0.02 |
|  |  | 37-80 |  |  | 37-77 |  |  | 37-83 | |  |  |  |  |  |  |  |
|  |  |  |  |  |  |  |  |  | |  |  |  |  |  |  |  |
| **BRIEF MI** | 33 | 52.39 (11.65) | [50.47,  54.85] | 50 | 53.40 (10.35) | [50.46,  56.34] | 34 | 50.76 (11.32) | | [46.81,  54.72] | 0.49 | 0.01 | 0.60 | 0.01 | 0.74 | 0.00 |
|  |  | 33-82 |  |  | 35-76 |  |  | 35-85 | |  |  |  |  |  |  |  |
|  |  |  |  |  |  |  |  |  | |  |  |  |  |  |  |  |
| Initiate | 19 | 52.84 (11.29) | [47.40,  58.28] | 35 | 55.31 (11.32) | [51.43,  59.20] | 34 | 49.53 (11.18) | | [45.63,  53.43] | 0.16 | 0.05 | 0.52 | 0.01 | 0.60 | 0.01 |
|  |  | 37-76 |  |  | 37-79 |  |  | 35-69 | |  |  |  |  |  |  |  |
|  |  |  |  |  |  |  |  |  | |  |  |  |  |  |  |  |
| Working Memory | 19 | 56.42 (12.47) | [50.41,  62.43] | 35 | 57.20 (13.39) | [52.60,  61.80] | 34 | 52.12 (9.75) | | [48.71,  55.52] | 0.27 | 0.03 | 0.51 | 0.01 | 0.91 | 0.00 |
|  |  | 39-89 |  |  | 38-89 |  |  | 38-71 | |  |  |  |  |  |  |  |
|  |  |  |  |  |  |  |  |  | |  |  |  |  |  |  |  |
| Plan/Organize | 19 | 56.47 (13.01) | [50.20,  62.74] | 35 | 54.51 (10.81) | [50.80,  58.23] | 34 | 49.59 (9.63) | | [46.23,  52.95] | 0.10 | 0.06 | 0.10 | 0.06 | 0.38 | 0.02 |
|  |  | 38-86 |  |  | 39-78 |  |  | 39-74 | |  |  |  |  |  |  |  |
|  |  |  |  |  |  |  |  |  | |  |  |  |  |  |  |  |
| Organise Material | 19 | 48.89 (8.35) | [44.87,  52.92] | 35 | 50.23 (10.49) | [46.62,  53.83] | 34 | 48.35 (7.87) | | [45.60,  51.10] | 0.84 | 0.01 | 0.76 | 0.00 | 0.89 | 0.00 |
|  |  | 37-71 |  |  | 34-69 |  |  | 34-67 | |  |  |  |  |  |  |  |
|  |  |  |  |  |  |  |  |  | |  |  |  |  |  |  |  |
| **Delis-Kaplan**  **Tower Time** | 38 | 590.47 (152.97) | [549.19,  640.75] | 62 | 547.44 (139.39) | [512.04,  582.83] | 44 | 559.86 (154.66) | | [512.84,  606.89] | 0.33 | 0.02 | 0.44 | 0.01 | 0.26 | 0.01 |
|  |  | 276-979 |  |  | 228-861 |  |  | 265-872 | |  |  |  |  |  |  |  |
|  |  |  |  |  |  |  |  |  | |  |  |  |  |  |  |  |
| *Note.* CYA-SCD-SCI+= children and young adults living with sickle cell disease with silent cerebral infarcts; CYA-SCD-SCI-= children and young adults living with sickle cell disease without silent cerebral infarcts, CYA-TD= children and young adults who are typically developing, SES= Socioeconomic status, SpO_2_= Oxygen saturation, CaO_2_= arterial oxygen content, FSIQ= Full Scale IQ, VCI= Verbal Comprehension Index, PRI= Perceptual Reasoning Index, WMI= Working Memory Index, PSI= Processing Speed Index, BRIEF= Behaviour Rating Inventory of Executive Function, BRIEF GEC= Global Executive Composite, BRIEF BRI= Regulation Index, BRIEF MI= Metacognition Index.  Results are given in mean (±SD) and range. *p value for Kruskal-Wallis test. *p^1^=* group comparison for CYA-SCD-SCI+ and CYA-SCD-SCI- and CYA-TD controlled for confounding variables (age, sex, SES). *p^2^=* group comparison for CYA-SCD-SCI+ and CYA-TD controlled for confounding variables (age, sex, SES). *p^3^=* group comparison for CYA-SCD-SCI+ and CYA-SCD-SCI- controlled for confounding variables (age, sex, SES). | | | | | | | | | | | | | | | | |

| **Supplement Table S2** | | | | | | | | | | | | | | |  |
| --- | --- | --- | --- | --- | --- | --- | --- | --- | --- | --- | --- | --- | --- | --- | --- |
| *Simple linear regression of all scaled scores for children and young adults living with sickle cell disease and for typically developing children and young adults* | | | | | | | | | | | | |  | | |
|  |  |  |  |  |  |  |  |  |  |  |  |  | | |  |
| **Cognitive Variables** | | **CYA-SCD** | | | | | **CYA-TD** | | | | | | |  | |
|  |  |  |  |  |  |  |  |  |  |  |  |  |  |  | |
|  | *F* | *p* | *R^2^* | *Unst. B* | *Std. Beta* | *CI 95%* | *F* | *p* | *R^2^* | *Unst. B* | *Std. Beta* | *CI 95%* | | |  |
| **Wechsler Cognitive Scores** | | |  |  |  |  |  |  |  |  |  |  | | |  |
| **VCI** | 22.49 | <0.001 | 0.25 | 1.48 | 0.50 | [.855, 2.098] | 8.88 | 0.004 | 0.15 | 0.80 | 0.39 | [.262, 1.346] | | |  |
| Similarities | 8.58 | 0.005 | 0.11 | 0.20 | 0.34 | [.063, .331] | 5.00 | 0.03 | 0.09 | 0.12 | 0.30 | [.012, .225] | | |  |
| Vocabulary | 18.77 | <.001 | 0.14 | 0.02 | 0.37 | [.010, .028] | 8.68 | 0.005 | 0.15 | 0.02 | 0.38 | [.005, .026] | | |  |
| **PRI** | 6.15 | 0.02 | 0.08 | 0.67 | 0.29 | [.131, 1.214] | 5.91 | 0.02 | 0.13 | 0.61 | 0.32 | [.105, 1.105] | | |  |
| Block Design | 2.02 | 0.16 | 0.03 | 0.08 | 0.17 | [-.032, .193] | 12.03 | 0.001 | 0.18 | 0.17 | 0.44 | [.072, .271] | | |  |
| Matrix Reasoning | 3.99 | 0.05 | 0.03 | 0.01 | 0.18 | [.000, .018] | 1.97 | 0.17 | 0.04 | 0.01 | 0.19 | [-.003, .018] | | |  |
| **WMI** | 3.80 | 0.05 | 0.03 | 0.04 | 0.18 | [-.001, .087] | 7.38 | 0.009 | 0.13 | 0.07 | 0.36 | [.018, .121] | | |  |
| Digit Span | 3.78 | 0.05 | 0.03 | 0.01 | 0.18 | [.000, .016] | 2.08 | 0.16 | 0.04 | 0.01 | 0.01 | [-.003, .019] | | |  |
| Arithmetic | 1.10 | 0.30 | 0.01 | 0.005 | 0.10 | [-.005, .015] | 2.50 | 0.12 | 0.05 | 0.01 | 0.01 | [-.002, .020] | | |  |
| **PSI** | 0.51 | 0.48 | 0.004 | 0.02 | 0.07 | [-.028, .059] | 2.97 | 0.09 | 0.06 | 0.05 | 0.03 | [-.008, .108] | | |  |
| Coding | 2.88 | 0.09 | 0.02 | 0.01 | 0.004 | [-.001, .016] | 4.78 | 0.03 | 0.09 | 0.01 | 0.29 | [.001, .025] | | |  |
| Symbol Search | 0.15 | 0.70 | 0.001 | 0.002 | 0.04 | [-.008, .012] | 0.00 | 0.99 | 0.00 | -0.00 | -0.001 | [-.012, .012] | | |  |
| Cancellation | 3.31 | 0.07 | 0.03 | -0.01 | -0.17 | [-.022, .001] | 1.19 | 0.28 | 0.02 | 0.01 | 0.01 | [-.006, .019] | | |  |
| **Executive Function Scores** | | |  |  |  |  |  |  |  |  |  |  | | |  |
| **BRIEF GEC** | 0.10 | 0.76 | 0.001 | -0.01 | -0.03 | [-.044, .032] | 3.70 | 0.06 | 0.07 | 0.09 | 0.51 | [.037, .135] | | |  |
| **BRIEF BRI** | 2.39 | 0.13 | 0.03 | -0.03 | -0.16 | [-.068, .008] | 7.25 | 0.01 | 0.16 | 0.07 | 0.41 | [.017, .122] | | |  |
| Inhibit | 0.12 | 0.73 | 0.002 | -0.01 | -0.04 | [-.057, .040] | 15.68 | <0.001 | 0.30 | 0.09 | 0.55 | [.044, .137] | | |  |
| Shift | 0.01 | 0.93 | 0.000 | 0.002 | 0.01 | [-.047, .052] | 3.51 | 0.07 | 0.09 | 0.05 | 0.29 | [-.004, .102] | | |  |
| Emotional Control | 1.27 | 0.27 | 0.02 | -0.03 | -0.14 | [-.078, .022] | 2.72 | 0.11 | 0.07 | 0.05 | 0.26 | [-.012, .117] | | |  |
| **BRIEF MI** | 0.30 | 0.59 | 0.003 | 0.01 | 0.06 | [-.028, .049] | 15.54 | <0.001 | 0.30 | 0.10 | 0.54 | [.047, .147] | | |  |
| Initiate | 0.06 | 0.81 | 0.001 | 0.01 | 0.03 | [-.042, .053] | 12.13 | 0.001 | 0.25 | 0.08 | 0.50 | [.035, .133] | | |  |
| Working Memory | 1.76 | 0.19 | 0.03 | -0.04 | -0.16 | [-.088, .018] | 14.82 | <0.001 | 0.29 | 0.08 | 0.54 | [.039, .125] | | |  |
| Plan/Organize | 0.97 | 0.33 | 0.02 | -0.02 | -0.12 | [-.070, .024] | 6.66 | 0.01 | 0.15 | 0.06 | 0.39 | [.013, .110] | | |  |
| Organise Material | 0.22 | 0.64 | 0.003 | 0.01 | 0.06 | [-.032, .051] | 1.97 | 0.17 | 0.05 | 0.03 | 0.23 | [-.013, .069] | | |  |
| **Delis-Kaplan Tower Time** | 0.30 | 0.59 | 0.003 | -0,13 | -0.05 | [-.610, .346] | 3.70 | 0.06 | 0.07 | -0.62 | -0.27 | [-1.276, .028] | | |  |
| *Note.* CYA-SCD= children and young adults living with sickle cell disease; CYA-TD= children and young adults who are typically developing, Unst.= unstandardised, Std.= standardised, FSIQ= Full Scale IQ, VCI= Verbal Comprehension Index, PRI= Perceptual Reasoning Index, WMI= Working Memory Index, PSI= Processing Speed Index, BRIEF= Behaviour Rating Inventory of Executive Function, BRIEF GEC= Global Executive Composite, BRIEF BRI= Regulation Index, BRIEF MI= Metacognition Index. | | | | | | | | | | | | | | | |

| **Supplement Table S3** |  |  |  |  |
| --- | --- | --- | --- | --- |
| *Results for the rotational analysis for scaled scores for children and young adults living with sickle cell disease and for typically developing children and young adults* | | | | |
| **Cognitive Variables** | **Relationship between MA x variables (unrotated)** | | **Zero trajectory** | **No Systematic relationship** |
|  | CYA-SCD | CYA-TD |  |  |
| **Wechsler Cognitive scores** |  |  |  |  |
| PRI Block Design | NS | Sig | × | ✓ |
| PSI Coding | NS | Sig | × | ✓ |
| **Executive Function scores** |  |  |  |  |
| **BRIEF GEC** | NS | Sig | ✓ (NS) | ✓ |
| **BRIEF BRI** | NS | Sig | × | ✓ |
| Inhibition | NS | Sig | ✓ (Sig) | × |
| **BRIEF MI** | NS | Sig | × | ✓ |
| Initiate | NS | Sig | ✓ (NS) | × |
| Working memory | NS | Sig | × | ✓ |
| Plan and organize | NS | Sig | × | ✓ |
| **Delis-Kaplan Tower Time** | NS | Sig | × | ✓ |
| *Note.* CYA-SCD= children and young adults living with sickle cell disease; CYA-TD= children and young adults who are typically developing, PRI= Perceptual Reasoning Index, PSI= Processing Speed Index, BRIEF= Behaviour Rating Inventory of Executive Function, BRIEF GEC= Global Executive Composite, BRIEF BRI= Regulation Index, BRIEF MI= Metacognition Index. NS= no-significant result (p > 0.05), Sig.= significant results (*p* < 0.05).  ✓ = yes, × = no.  Rotation analyses can only be conducted in variables where CYA-TD has a significant relationship with mental age, but CYA-SCD group does not show a significant relationship with mental age. | | | | |

| **Supplement Table S4**  *Simple linear regression of all scaled scores (SCI) for children and young adults living with sickle cell disease with or without silent cerebral infarcts and for typically developing children and young adults* | | | | | | | | | | | | | | | | | | | | | |
| --- | --- | --- | --- | --- | --- | --- | --- | --- | --- | --- | --- | --- | --- | --- | --- | --- | --- | --- | --- | --- | --- |
| **Cognitive Variables** | | **CYA-SCD** | | | | | | | | | | | | | | ***CYA-TD*** | | | | | |
|  |  | ***SCI+*** | | | | | | | ***SCI-*** | | | | | | |  |  |  |  |  |  |
|  | *F* | | *p* | *R^2^* | *Unst. B* | *Std. Beta* | *CI 95%* | *F* | | *p* | *R^2^* | *Unst. B* | *Std. Beta* | *CI 95%* | *F* | | *p* | *R^2^* | *Unst. B* | *Std. Beta* | *CI 95%* |
| **Wechsler Cognitive Scores** | | | | | | | | | | | | | | | | | | | | | |
| **VCI** | 4.41 | | 0.05 | 0.20 | 0.11 | 0.44 | [.00, .22] | 21.61 | | <0.001 | 0.40 | 0.14 | 0.63 | [.08, .20] | 6.58 | | 0.01 | 0.14 | 0.06 | 0.38 | [.01, .11] |
| Similarities | 2.81 | | 0.11 | 0.14 | 0.02 | 0.37 | [-.01, .04] | 7.94 | | 0.01 | 0.19 | 0.02 | 0.44 | [.01, .03] | 3.73 | | 0.06 | 0.09 | 0.01 | 0.30 | [.00, .02] |
| Vocabulary | 3.37 | | 0.07 | 0.08 | 0.02 | 0.28 | [-.002, .03] | 14.96 | | <0.001 | 0.19 | 0.02 | 0.44 | [.01, .03] | 5.94 | | 0.02 | 0.13 | 0.02 | 0.36 | [.00, .03] |
| **PRI** | 1.07 | | 0.31 | 0.06 | 0.04 | 0.24 | [-.04, .12] | 5.96 | | 0.02 | 0.15 | 0.08 | 0.39 | [.01, .14] | 4.95 | | 0.03 | 0.11 | 0.05 | 0.34 | [.01, .10] |
| Block Design | 6.99 | | 0.02 | 0.28 | 0.27 | 0.53 | [.06, .49] | 0.18 | | 0.68 | 0.01 | 0.03 | 0.07 | [-.11, .17] | 8.14 | | 0.01 | 0.16 | 0.16 | 0.40 | [.05, .25] |
| Matrix Reasoning | 0.001 | | 0.97 | 0 | -0.004 | -0.01 | [-.23, .22] | 5.56 | | 0.02 | 0.08 | 0.16 | 0.29 | [.02, .29] | 2.00 | | 0.16 | 0.04 | 0.10 | 0.21 | [-.04, .24] |
| **WMI** | 2.07 | | 0.16 | 0.05 | 0.06 | 0.22 | [-.02, .14] | 2.33 | | 0.13 | 0.04 | 0.04 | 0.19 | [-.01, .10] | 8.34 | | 0.01 | 0.18 | 0.07 | 0.42 | [.03, .15] |
| Digit Span | 5.71 | | 0.02 | 0.13 | 0.24 | 0.35 | [.04, .45] | 1.03 | | 0.33 | 0.02 | 0.06 | 0.13 | [-.06, .18] | 3.26 | | 0.08 | 0.07 | 0.13 | 0.26 | [-.02, .28] |
| Arithmetic | 1.04 | | 0.31 | 0.03 | 0.11 | 0.16 | [-.11, .33] | 0.86 | | 0.36 | 0.02 | 0.07 | 0.12 | [-.08, .22] | 1.55 | | 0.22 | 0.04 | 0.10 | 0.19 | [-.06, .25] |
| **PSI** | 2.69 | | 0.11 | 0.06 | 0.73 | 0.25 | [-.17, 1.63] | 0.11 | | 0.74 | 0.002 | 0.11 | 0.04 | [-.54, .76] | 2.99 | | 0.09 | 0.06 | 0.70 | 0.25 | [-.12, 1.51] |
| Coding | 4.21 | | 0.05 | 0.10 | 0.01 | 0.31 | [.00, .03] | 1.17 | | 0.28 | 0.02 | 0.01 | 0.14 | [-.01, .02] | 3.88 | | 0.06 | 0.09 | 0.01 | 0.30 | [.00, .03] |
| Symbol Search | 1.25 | | 0.27 | 0.03 | 0.13 | 0.17 | [-.11, .37] | 0.04 | | 0.85 | 0.001 | 0.01 | 0.03 | [-.13, .15] | 0.46 | | 0.50 | 0.01 | 0.05 | 0.10 | [-.11, .21] |
| Cancellation | 6.74 | | 0.01 | 0.14 | -0.32 | -0.38 | [-.57, -.07] | 0.09 | | 0.77 | 0.001 | -0.02 | -0.04 | [-.19, .14] | 2.39 | | 0.13 | 0.05 | 0.13 | 0.23 | [-.04, .30] |
| **Executive Function Scores** | | | | | | | | | | | | | | | | | | | | | |
| **BRIEF GEC** | 0.14 | | 0.71 | 0.01 | 0.01 | 0.07 | [-.06, .09] | 0.61 | | 0.44 | 0.01 | -0.02 | -0.11 | [-.07, .03] | 6.27 | | 0.02 | 0.18 | 0.07 | 0.42 | [.01, .12] |
| **BRIEF BRI** | 0.01 | | 0.93 | 0 | -0.04 | -0.02 | [-.88, .80] | 3.28 | | 0.08 | 0.07 | -0.50 | -0.26 | [-1.06, .06] | 2.45 | | 0.13 | 0.08 | 0.05 | 0.28 | [-.01, .11] |
| Inhibit | 0.67 | | 0.42 | 0.04 | 0.03 | 0.20 | [-.04, .10] | 0.58 | | 0.45 | 0.02 | -0.02 | -0.13 | [-.08, .04] | 9.10 | | 0.01 | 0.22 | 0.95 | 0.47 | [.31, 1.59] |
| Shift | 0.16 | | 0.70 | 0.01 | 0.21 | 0.10 | [-.91, 1.34] | 0.26 | | 0.61 | 0.01 | -0.19 | -0.09 | [-.95, .57] | 1.70 | | 0.20 | 0.05 | 0.46 | 0.23 | [-.26, 1.17] |
| Emotional Control | 0.39 | | 0.54 | 0.02 | -0.42 | -0.15 | [-1.85, 1.0] | 1.50 | | 0.23 | 0.05 | -0.43 | -0.21 | [-1.14, .28] | 1.19 | | 0.28 | 0.04 | 0.46 | 0.19 | [-.39, 1.30] |
| **BRIEF MI** | 0.43 | | 0.52 | 0.01 | 0.025 | 0.12 | [-.05, .10] | 0.002 | | 0.96 | 0 | -0.001 | -0.01 | [-.05, .05] | 10.54 | | 0.003 | 0.27 | 0.09 | 0.52 | [.03, .15] |
| Initiate | 0.03 | | 0.87 | 0.002 | -0.01 | -0.04 | [-.11, .09] | .002 | | 0.97 | 0 | -0.001 | -0.01 | [-.06, .06] | 6.96 | | 0.01 | 0.19 | 0.08 | 0.44 | [.02, .14] |
| Working Memory | 0.98 | | 0.34 | 0.05 | -0.05 | -0.23 | [-.16, .06] | 1.31 | | 0.26 | 0.04 | -0.04 | -0.20 | [-.11, .03] | 7.69 | | 0.01 | 0.21 | 0.07 | 0.46 | [.02, .12] |
| Plan/Organize | 0.46 | | 0.51 | 0.03 | -0.04 | -0.16 | [-.15, .08] | 2.08 | | 0.16 | 0.06 | -0.04 | -0.24 | [-.10, .02] | 4.36 | | 0.05 | 0.13 | 0.05 | 0.36 | [.00, .11] |
| Organise Material | 0.04 | | 0.85 | 0.002 | 0.08 | 0.05 | [-.80, .95] | 0.07 | | 0.80 | 0.002 | 0.09 | 0.04 | [-.59, .76] | 1.38 | | 0.25 | 0.04 | 0.31 | 0.20 | [-.22, .83] |
| **Delis-Kaplan Tower Time** | 7.33 | | 0.01 | 0.20 | 0.08 | 0.45 | [.02, .13] | 3.09 | | 0.09 | 0.08 | -0.69 | -0.28 | [-1.48, .11] | 0.31 | | 0.58 | 0.01 | 0.16 | 0.07 | [-.41, .72] |
| *Note.* CYA-SCD-SCI+= children and young adults with living sickle cell disease with silent cerebral infarcts; CYA-SCD-SCI-= children and young adults living with sickle cell disease without silent cerebral infarcts, CYA-TD= children and young adults who are typically developing, Unst.= unstandardised, Std.= standardised, FSIQ= Full Scale IQ, VCI= Verbal Comprehension Index, PRI= Perceptual Reasoning Index, WMI= Working Memory Index, PSI= Processing Speed Index, BRIEF= Behaviour Rating Inventory of Executive Function, BRIEF GEC= Global Executive Composite, BRIEF BRI= Regulation Index, BRIEF MI= Metacognition Index. | | | | | | | | | | | | | | | | | | | | | |

| **Supplement Table S5**  *Simple linear regression of all raw scores for children and young adults living with sickle cell disease and for typically developing children*  *and young adults* | | | | | | | | | | | | | |
| --- | --- | --- | --- | --- | --- | --- | --- | --- | --- | --- | --- | --- | --- |
| ***Cognitive Variables*** | **CYA-SCD** | | | | | | **CYA-TD** | | | | | |  |
|  |  |  |  |  |  |  |  |  |  |  |  |  |  |
|  | *F* | *p* | *R^2^* | *Unst. B* | *Std. Beta* | *CI 95%* | *F* | *p* | *R^2^* | *Unst. B* | *Std. Beta* | *CI 95%* |  |
| **WISC VCI** |  |  |  |  |  |  |  |  |  |  |  |  |  |
| Similarities | 12.41 | 0.002 | 0.33 | 2.32 | 0.60 | [.96, 3.69] | 4.92 | 0.04 | 0.20 | 0.66 | 0.50 | [.06, 2.87] |  |
| Vocabulary | 64.36 | <.001 | 0.79 | 3.24 | 0.89 | [2.39, 4.10] | 2.52 | 0.13 | 0.09 | 1.91 | 0.38 | [-.65, 4.48] |  |
| **WISC PRI** |  |  |  |  |  |  |  |  |  |  |  |  |  |
| Block Design | 4.46 | 0.05 | 0.13 | 2.86 | 0.40 | [.06, 5.65] | 6.58 | 0.02 | 0.26 | 3.37 | 0.55 | [.57, 6.17] |  |
| Matrix Reasoning | 8.27 | 0.01 | 0.33 | 0.92 | 0.57 | [.25, 1.60] | 0.40 | 0.54 | -0.04 | 0.40 | 0.16 | [-.94, 1.74] |  |
| **WISC WMI** |  |  |  |  |  |  |  |  |  |  |  |  |  |
| Digit Span | 3.56 | 0.07 | 0.07 | 1.74 | 0.27 | [-.12, 3.61] | 0.04 | 0.85 | -0.06 | 0.06 | 0.28 | [-.53, .64] |  |
| Arithmetic | 64.35 | <.001 | 0.73 | 1.79 | 0.85 | [1.33, 2.25] | 0.05 | 0.82 | -0.08 | 0.16 | 0.07 | [-1.34, 1.66] |  |
| **WISC PSI** |  |  |  |  |  |  |  |  |  |  |  |  |  |
| Coding | 9.79 | 0.003 | 0.18 | 2.61 | 0.42 | [.93, 4.29] | 2.47 | 0.14 | 0.08 | 1.92 | 0.38 | [-1.08, 7.12] |  |
| Symbol Search | 8.13 | 0.01 | 0.12 | 4.59 | 0.37 | [1.36, 7.82] | 4.18 | 0.06 | 0.17 | 1.49 | 0.47 | [-.06, 3.05] |  |
| Cancellation | 0.29 | 0.61 | 0.01 | 1.00 | 0.08 | [-2.87, 4.87] | 0.40 | 0.54 | -0.04 | 1.97 | 0.16 | [-4.65, 8.59] |  |
| **Executive Function Scores** | |  |  |  |  |  |  |  |  |  |  |  |  |
| **BRIEF GEC** | 2.47 | 0.12 | 0.02 | -0.85 | -0.16 | [-1.91, -.16] | 7.20 | 0.01 | 0.18 | 2.24 | 0.45 | [.56, 3.92] |  |
| **BRIEF BRI** | 0.09 | 0.76 | -0.01 | -0.07 | -0.03 | [-.54, .40] | 10.08 | 0.004 | 0.23 | 1.30 | 0.51 | [.46, 2.13] |  |
| Inhibit | 5.59 | 0.02 | 0.05 | -0.19 | -0.24 | [-0.35, -.03] | 3.41 | 0.08 | 0.07 | 0.21 | 0.32 | [-.02, .44] |  |
| Shift | 12.41 | <.001 | 0.14 | -0.25 | -0.37 | [-.39, -.11] | 0.18 | 0.68 | -0.03 | -0.04 | -0.08 | [-.22, .14] |  |
| Emotional Control | 2.93 | 0.09 | 0.04 | -0.18 | -0.19 | [-.40, .03] | 1.83 | 0.19 | 0.03 | 0.28 | 0.24 | [.19, -.15] |  |
| **BRIEF MI** | 6.33 | 0.01 | 0.08 | -0.92 | -0.27 | [-1.66, -.19] | 3.39 | 0.08 | 0.07 | 0.84 | 0.32 | [-.09, 1.77] |  |
| Initiate | 0.28 | 0.06 | -0.01 | 0.04 | 0.05 | [-.11, .19] | 14.94 | <0.001 | 0.32 | 0.38 | 0.58 | [.18, .58] |  |
| Working Memory | 21.84 | <.001 | 0.22 | -0.43 | -0.47 | [-.61, -.25] | 0.54 | 0.47 | -0.02 | 0.07 | 0.14 | [-.13, .28] |  |
| Plan/Organize | 13.52 | <.001 | 0.15 | -0.40 | -0.38 | [-.62, -.81] | 0.001 | 0.98 | -0.03 | 0.004 | 0.004 | [-.31, .33] |  |
| Organise Material | 7.07 | 0.01 | 0.06 | 0.20 | 0.27 | [.05, .34] | 9.33 | 0.005 | 0.22 | 0.31 | 0.49 | [.11, .57] |  |
| **Delis-Kaplan Tower Time** | 0.48 | 0.49 | 0.01 | -0.03 | -0.08 | [-.11, .05] | 0.16 | 0.697 | -0.05 | -0.02 | -0.10 | [-.15, .10] |  |
|  |  |  |  |  |  |  |  |  |  |  |  |  |  |
| *Note.* CYA-SCD= children and young adults living with sickle cell disease; CYA-TD= children and young adults who are typically developing, Unst.= unstandardised, Std.= standardised, FSIQ= Full Scale IQ, VCI= Verbal Comprehension Index, PRI= Perceptual Reasoning Index, WMI= Working Memory Index, PSI= Processing Speed Index, BRIEF= Behaviour Rating Inventory of Executive Function, BRIEF GEC= Global Executive Composite, BRIEF BRI= Regulation Index, BRIEF MI= Metacognition Index. | | | | | | | | | | | | |  |

| **Supplement Table S6**  *Developmental trajectories for raw scores for children and young adults living with sickle cell disease with or without silent cerebral infarcts and for*  *typically developing children and young adults.* | | | | | | | | | | | |
| --- | --- | --- | --- | --- | --- | --- | --- | --- | --- | --- | --- |
| **Variable** | **Slope: Cognitive variable = (Intercept for group) + (age* gradient)** | | | **Delay at onset** | ***p*** | ***CI 95%*** | **Rate of development** | ***p*** | ***CI 95%*** |  |  |
|  |  |  |  |  |  |  |  |  |  |  |  |
|  | **CYA-SCD** | **CYA-TD** | | **CYA-SCD compared with CYA-TD** | | | | | |  |  |
| **Wechsler Cognitive Scores (WISC)** |  |  |  |  |  |  |  |  |  |  |  |
| **VCI** |  |  | |  |  |  |  |  |  |  |  |
| Similarities | 6.33 + age*2.44 | 16.57 + age*1.02 | | 10.23 | 0.10 | [-1.95, 22.42] | 2.38 | 0.15 | [-3.37, .54] |  |  |
| Symbol Search | 15.59 + age*4.27 | 15.25 + age*1.55 | | -0.34 | 0.99 | [-37.97, 37.30] | 2.75 | 0.35 | [-8.44, 3.00] |  |  |
| **Executive Function Scores** |  |  |  |  |  |  |  |  |  |  |  |
| **BRIEF GEC** | 121. 34 + age*-0.85 | 84.26 + age*2.24 | | -37.07 | 0.002 | [-60.16, -13.98] | -0.38 | 0.004 | [1.04, 5.14] |  |  |
| **BRIEF BRI** | 44.43 + age*-0.73 | 31.28 + age*1.30 | | -13.15 | 0.014 | [-23.58, -2.72] | -0.5 | 0.004 | [0.44, 2.29] |  |  |
| Inhibit | 15.06 + age*-0.19 | 11.22 + age*0.21 | | -3.85 | 0.027 | [-7.22, -0.45] | -0.9 | 0.016 | [.098, .701] |  |  |
| **BRIEF MI** |  |  | |  |  |  |  |  |  |  |  |
| Initiate | 12.67 + age*0.04 | 7.65 + age*0.44 | | -5.02 | 0.002 | [-8.12, -1.92] | 0.09 | 0.016 | [0.06, .62] |  |  |
| Organise Materials | 10.04 + age*0.20 | 7.59 + age*0.34 | | -2.45 | 0.13 | [-5.61, 0.71] | 0.57 | 0.31 | [-.14, .43] |  |  |

*Note.* CYA-SCD= children and young adults living with sickle cell disease, CYA-TD= children and young adults who are typically developing, VCI= Verbal Comprehension Index, BRIEF= Behavior Rating Inventory of Executive Function, BRIEF GEC= Global Executive Composite, BRIEF BRI= Regulation Index, BRIEF MI= Metacognitive Index.

Delay at onset = intercept of CYA-TD group – Intercept of CYA-SCD group; rate of development = gradient of CYA-SCD group/gradient of CYA-TD group.

| **Supplement table S7**  *Results for the rotational analysis for raw scores for children and young adults with sickle cell disease and for typically developing children and young adults* |
| --- |

| **Cognitive Variable** | **Relationship between MA x variable (unrotated)** | | **Zero trajectory** | **No Systematic relationship** |
| --- | --- | --- | --- | --- |
|  | CYA-SCD | CYA-TD |  |  |
| **Executive Function scores** |  |  |  |  |
| **BRIEF GEC** | NS | Sig | × | ✓ |
| **BRIEF BRI** | NS | Sig | ✓(Sig.) | × |
| **BRIEF MI** |  |  |  |  |
| Initiate | NS | Sig | ✓ (Sig.) | × |
| *Note.* MA= mental age; CYA-SCD= children and young adults living with sickle cell disease; CYA-TD= children and young adults who are typically developing, BRIEF= Behaviour Rating Inventory of Executive Function, BRIEF GEC= Global Executive Composite, BRIEF BRI= Regulation Index, BRIEF MI= Metacognition Index. NS= no-significant result (p > 0.05), Sig.= significant results (*p* < 0.05).  ✓ = yes, × = no.  Rotation analyses can only be conducted in variables where CYA-TD has a significant relationship with mental age, but CYA-SCD group does not show a significant relationship with mental age. | | | | |

**Supplement Table S8**

*Developmental trajectory for BRIEF MI Initiate raw score for children and young adults living with sickle cell disease with or without silent cerebral infarcts and for typically developing children and young adults*

| **Variable** | | | | **Slope: Cognitive variable = (Intercept for group) + (age* gradient)** | | | | | | | | | | | | | | | | | | | |  |  |  |  |  |  |  |
| --- | --- | --- | --- | --- | --- | --- | --- | --- | --- | --- | --- | --- | --- | --- | --- | --- | --- | --- | --- | --- | --- | --- | --- | --- | --- | --- | --- | --- | --- | --- |
|  |  |  |  |  |  |  |  |  |  |  |  |  |  |  |  |  |  |  |  |  |  |  |  |  |  |  |  |  |  |  |
|  |  |  |  | **CYA-SCD** | | | | | | | | | | |  | | **CYA-TD** | | | | | | |  |  |  |  |  |  |  |
|  | | | | CYA-SCD-SCI+ | | | | | | CYA-SCD-SCI- | | | | |  | |  | | | |  | | |  |  |  |  |  |  |  |
| **BRIEF** Initiate | | | | 12.633 + age * .02 | | | | | | 13.275 + age * .022 | | | | | | | 7.785 + age * .384 | | | | | | |  |  |  |  |  |  |  |
|  |  | |  | | |  | |  | | |  | |  | | |  | | |  |  | | |  | | | |  |  |  |  |
|  | |  | | |  | |  | |  | | |  | |  | | | |  | |  | |  | | | |  | |  |  |  |
| **Variable** | | **Delay at onset CYA-SCD** | | | | | ***p*** | | | | | ***CI 95%*** | | | | | | **Rate of development for CYA-SCD** | | | | ***p*** | | | | | | ***CI 95%*** | |  |
|  |  |  |  |  |  |  |  |  |  |  |  |  |  |  |  |  |  |  |  |  |  |  |  |  |  |  |  |  |  |  |
|  |  | **CYA-SCD compared with CYA-TD** | | | | | | | | | | | | | | | | | | | | | | | | | | | |  |
|  | | SCI+ | | | SCI- | | SCI+ | | SCI- | | | SCI+ | | SCI- | | | | SCI+ | | SCI- | | SCI+ | | | | SCI- | | SCI+ | SCI- |  |
| **BRIEF MI** Initiate | | -4.84 | | | -5.49 | | 0.01 | | 0.71 | | | [9.98, 15.35] | | [-2.62, 3.84] | | | | 0.05 | | 0.06 | | 0.08 | | | | 0.99 | | [-0.25, 0.28] | [-0.32, 0.32] |  |
| *Note.* CYA-SCD-SCI+= children and young adults living with sickle cell disease with silent cerebral infarcts; CYA-SCD-SCI-= children and young adults living with sickle cell disease without silent cerebral infarcts, BRIEF= Behaviour Rating Inventory of Executive Function, MI = Metacognition Index.  Delay at onset = intercept of CYA-TD group – Intercept of CYA-SCD group; rate of development = gradient of CYA-SCD group/gradient of CYA-TD group. | | | | | | | | | | | | | | | | | | | | | | | | | | | | | |  |

| **Supplement Table S9**  *Simple Linear regression models for blood oxygenation measures and SES predicting cognitive variables in children and young adults living with sickle cell disease with silent cerebral infarcts* | | | | | | | |  |
| --- | --- | --- | --- | --- | --- | --- | --- | --- |
|  |  |  |  |  |  |  |  |  |
| **Measure** | **Cognitive Variables** | **CYA -SCD-SCI+** | | | | | |  |
|  |  |  |  |  |  |  |  |  |
|  |  | *F* | *p* | *R2* | *Unst. B* | *Std. Beta* | *CI 95%* |  |
| Haemoglobin |  |  |  |  |  |  |  |  |
|  | **PRI** | 7.51 | 0.01 | 0.29 | 0.36 | 0.54 | [.07, 0.54] |  |
| CaO_2_ |  |  |  |  |  |  |  |  |
|  | **VCI** | 5.01 | 0.04 | 0.22 | 3.0 | 0.47 | [.18, 5.81] |  |
|  |  |  |  |  |  |  |  |  |
|  | **PRI** | 10.52 | 0.01 | 0.37 | 2.52 | 0.61 | [.89, 4.15] |  |
| SES |  |  |  |  |  |  |  |  |
|  | **BRIEF BRI Inhibit** | 4.20 | 0.058 | 0.22 | -0.001 | -0.47 | [-0.001, 0] |  |
| *Note.* CYA-SCD-SCI+= children and young adults living with sickle cell disease and silent cerebral infarct, Unst. = unstandardised, Std.= standardised, CaO_2_= arterial oxygen content, SES= Socioeconomic status, VCI= Verbal Comprehension Index, PRI= Perceptual Reasoning Index, BRIEF= Behaviour Rating Inventory of Executive Function, BRIEF BRI= Regulation Index. | | | | | | | |  |
